# Supplementary material for: Structural insights into the DNA topoisomerase II of the African swine fever virus
Source: Nat Commun. 2024 May 30;15:4607. doi: 10.1038/s41467-024-49047-w (PMC11139879; doi:10.1038/s41467-024-49047-w)
Supplement: Supplementary file 1 — Supplementary Information [file 41467_2024_49047_MOESM1_ESM.pdf]

Supplementary information

Structural insights into DNA Topoisomerase II of the African Swine Fever Virus

J. Cong *et al.*

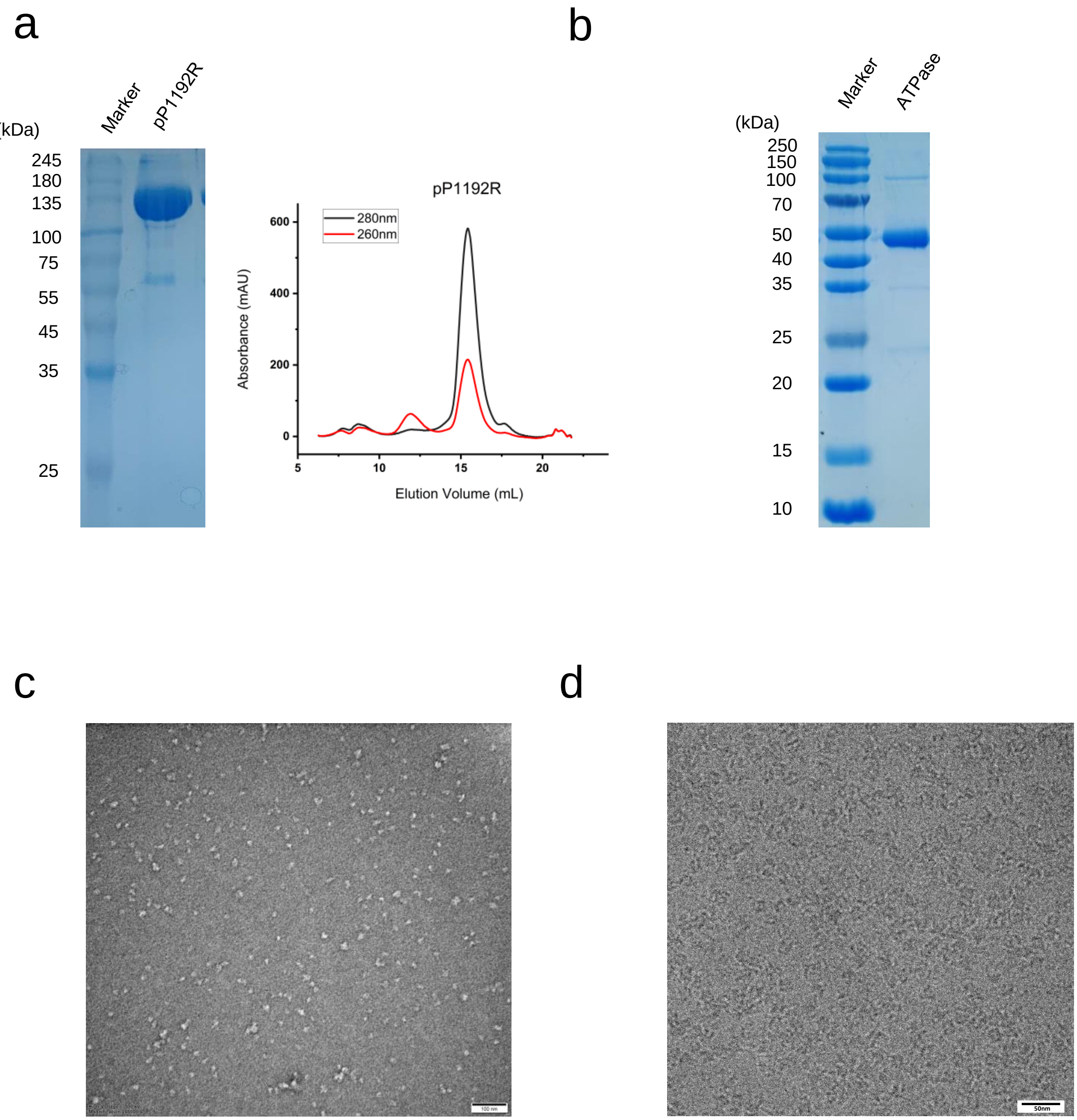

Supplementary Figure 1: Biochemical assays and negative staining/cryo-EM micrograph. a Gel filtration on a Superose 6 Increase size-exclusion column and SDS-PAGE analysis of the purified pP1192R. **b** SDS-PAGE analysis of the purified truncated ATPase domain. **c** A representative negative staining micrograph (apo full-length pP1192R). **d** A representative cryo-EM micrograph (apo full-length pP1192R). Source data are provided as a Source Data file.

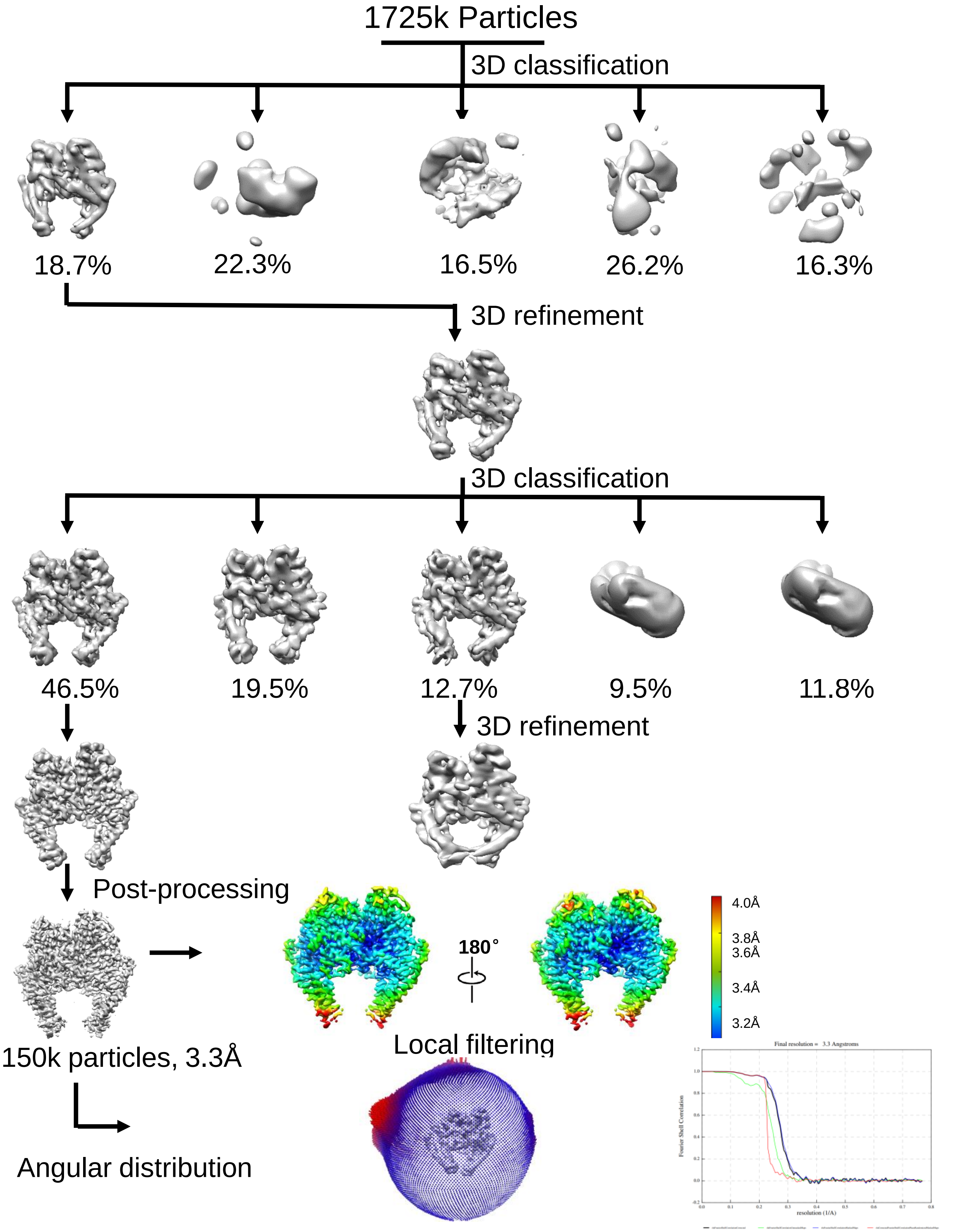

Supplementary Figure 2: Cryo-EM 3D-reconstruction of pP1192R$_{\text{Coil-open}}$. A schematic diagram illustrating the Cryo-EM data processing procedures for pP1192R$_{\text{Coil-open}}$. We extracted approximate 1725k particles, and after two rounds of 3D classification, obtained approximate 150k particles for further 3D reconstruction, with the final map reaching a resolution of 3.3Å. Local resolution, Euler angle distribution and the FSC curves for each reconstruction were demonstrated.

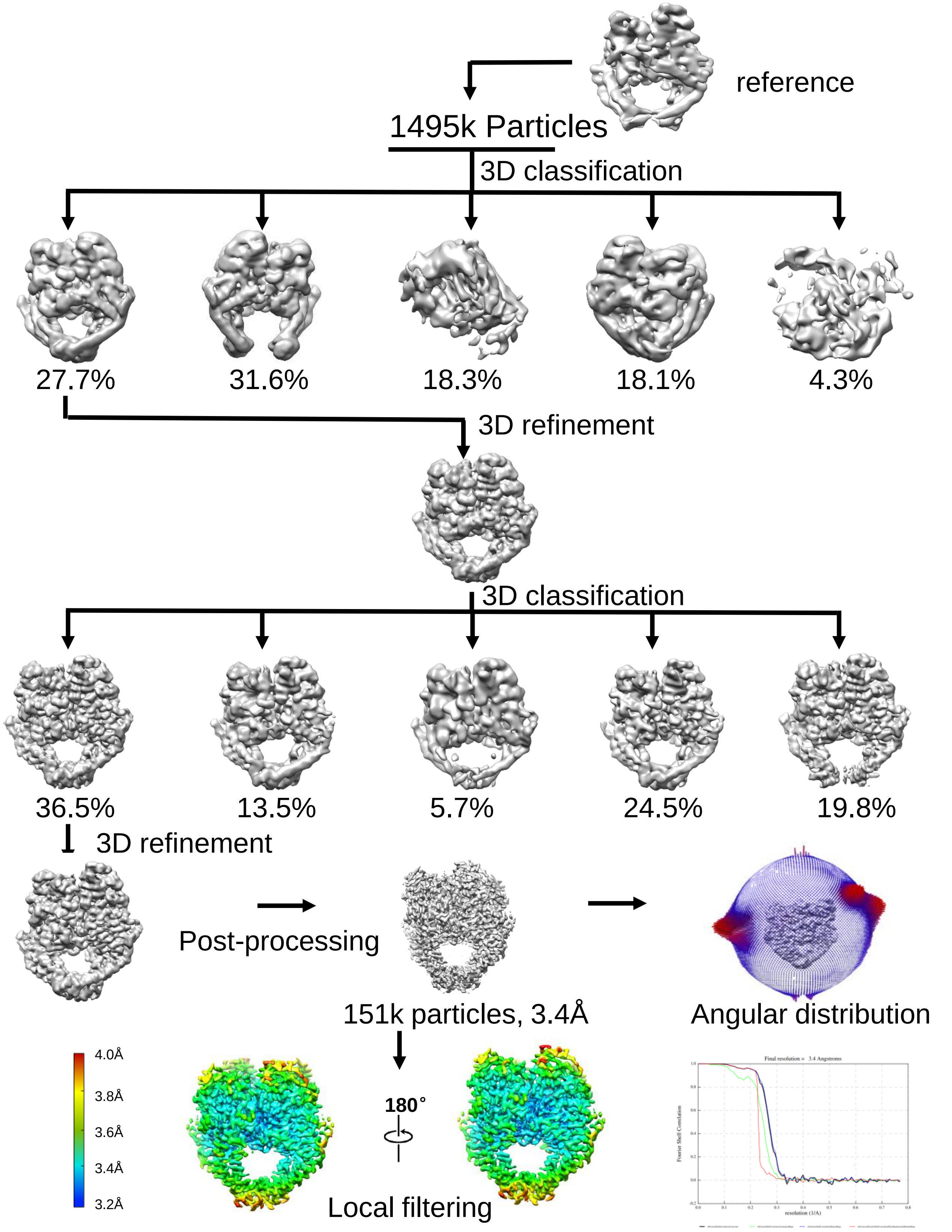

Supplementary Figure 3: Cryo-EM 3D-reconstruction of pP1192R$_{Close}$. A schematic diagram illustrating the Cryo-EM data processing procedures for pP1192R$_{Close}$. We extracted approximate 1495k particles, and after two rounds of 3D classification, obtained approximate 151k particles for further 3D reconstruction, with the final map reaching a resolution of 3.4Å. Local resolution, Euler angle distribution and the FSC curves for each reconstruction were demonstrated.

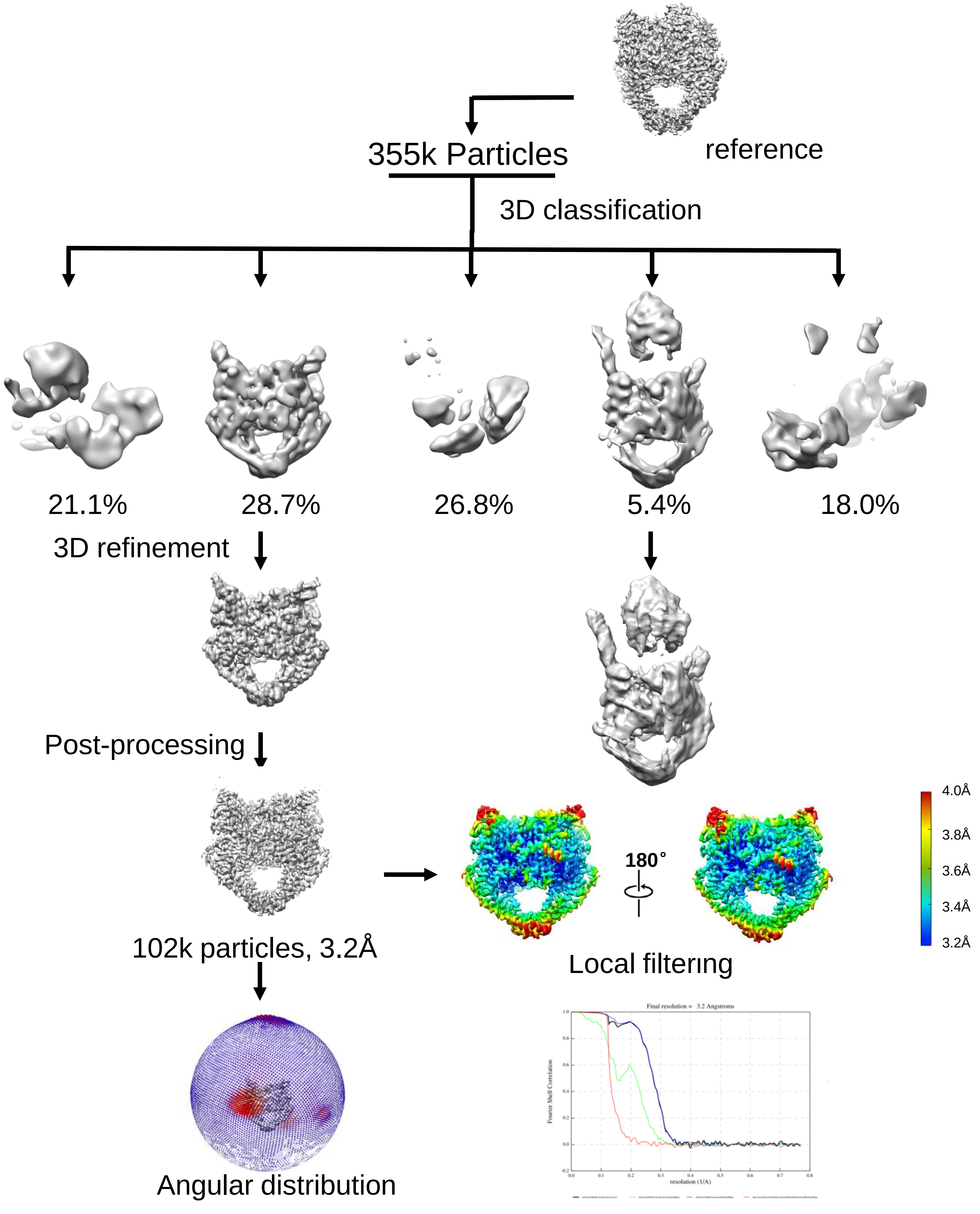

Supplementary Figure 4: Cryo-EM 3D-reconstruction of pP1192R$_{CD\text{-}DNA}$. A schematic diagram illustrating the Cryo-EM data processing procedures for pP1192R$_{CD\text{-}DNA}$. We extracted approximate 355k particles, and after one round of 3D classification, obtained approximate 102k particles for further 3D reconstruction, with the final map reaching a resolution of 3.2Å. Local resolution, Euler angle distribution and the FSC curves for each reconstruction were demonstrated.

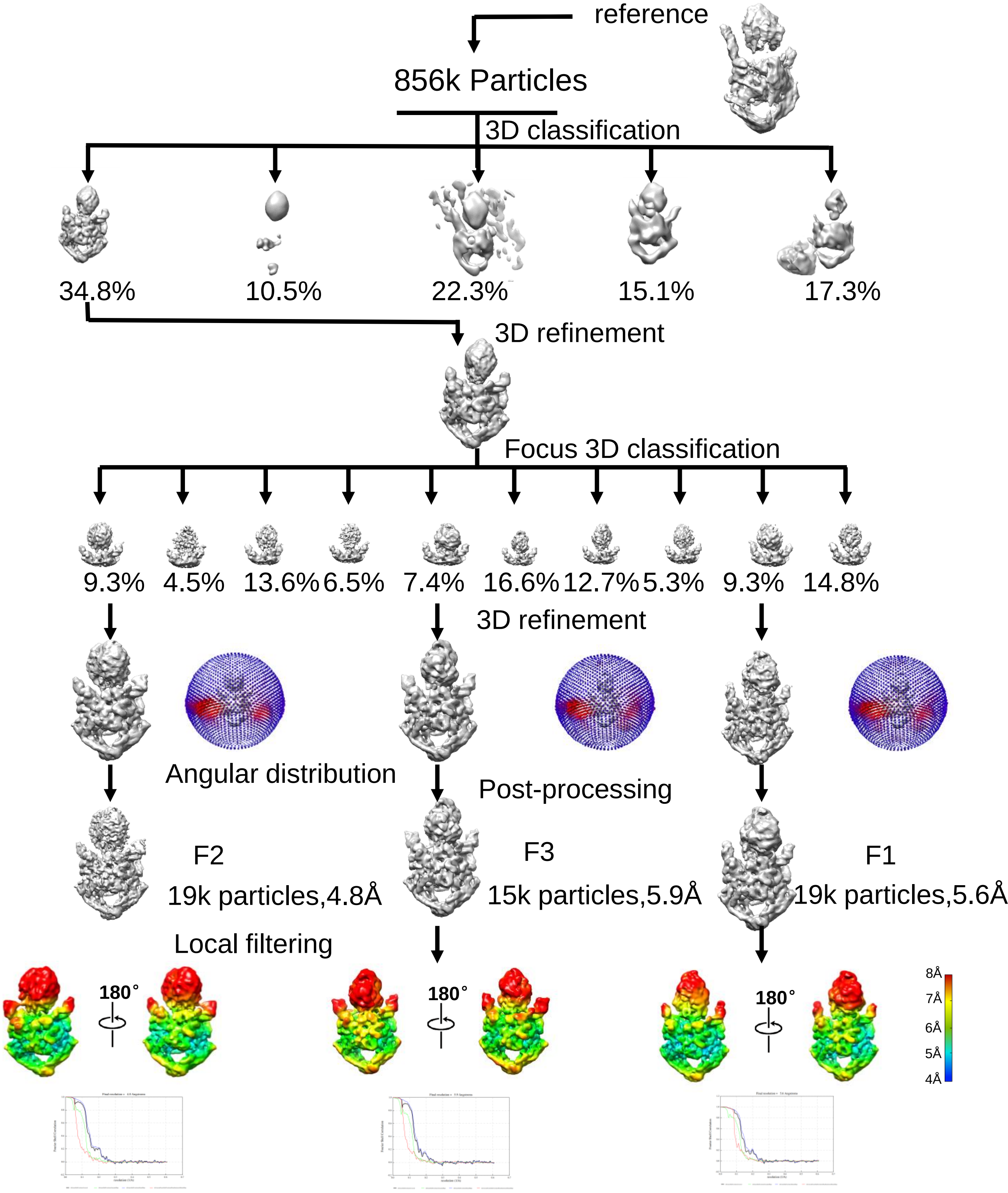

Supplementary Figure 5: Cryo-EM 3D-reconstruction of pP1192R$_{F1\text{-}3}$. A schematic diagram illustrating the Cryo-EM data processing procedures for pP1192R$_{F1\text{-}3}$. We extracted approximate 856k particles, and after one round of 3D classification and one round of focus 3D classification, obtained approximate 19k, 19k and 15k particles for further 3D reconstruction, with the final map reaching a resolution of pP1192R$_{F1\text{-}3}$ 5.6Å, 4.8Å and 5.9Å respectively. Local resolution, Euler angle distribution and the FSC curves for each reconstruction were demonstrated.

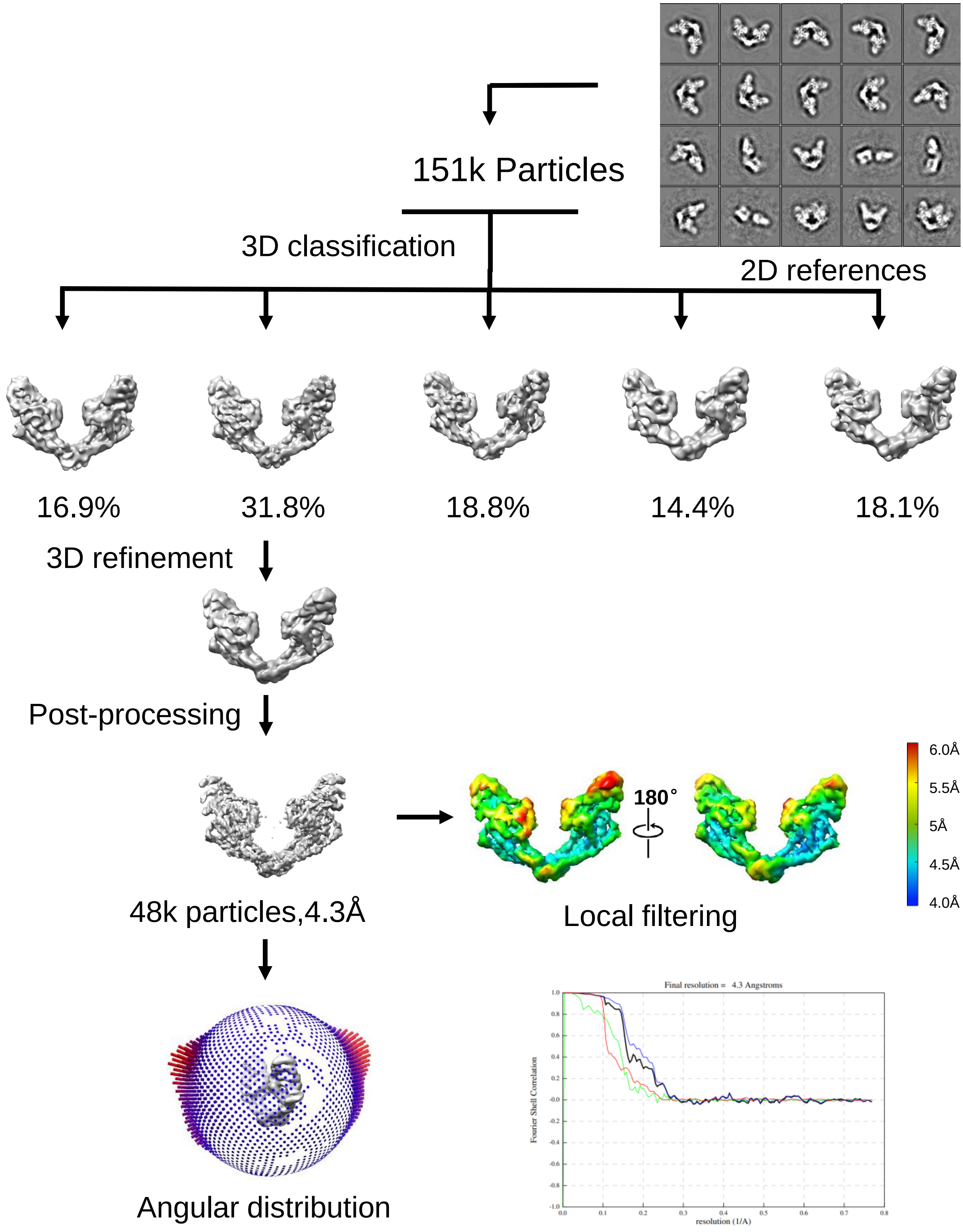

Supplementary Figure 6: Cryo-EM 3D-reconstruction of pP1192R$_{\text{WHD-open}}$. A schematic diagram illustrating the Cryo-EM data processing procedures for pP1192R$_{\text{WHD-open}}$. We extracted approximate 151k particles, and after one round of 3D classification, obtained approximate 48k particles for further 3D reconstruction, with the final map reaching a resolution of 4.3Å. Local resolution, Euler angle distribution and the FSC curves for each reconstruction were demonstrated.

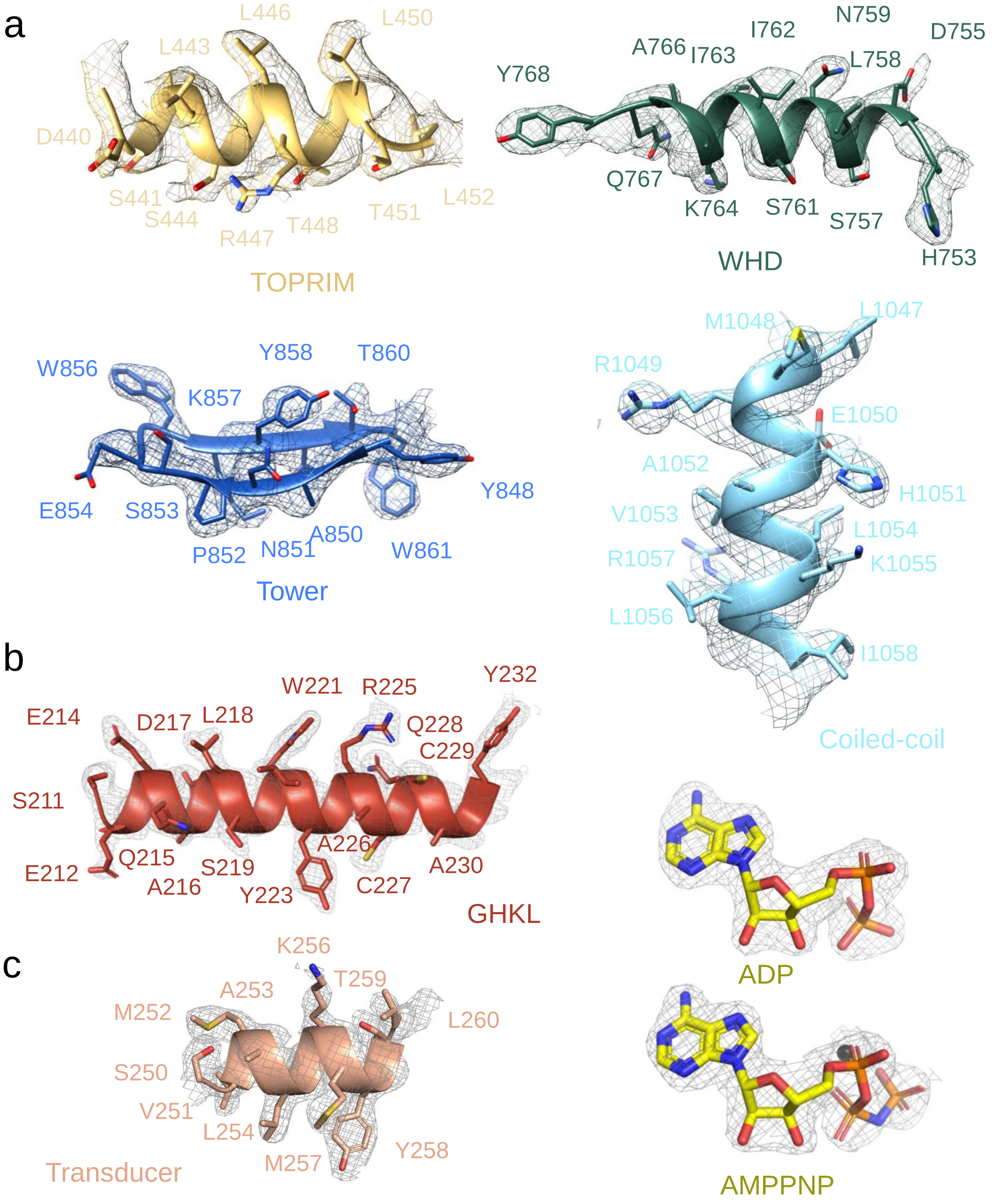

Supplementary Figure 7: Representative localized density maps of pP1192R reconstruction. Protein is shown in cartoon and colored as Fig. 2. Side chains are shown as sticks with oxygen, nitrogen and Phosphorus atoms shown in red, blue and orange, respectively. **a** Representative localized density maps of pP1192R$_{CD\text{-}DNA}$. **b** (2mFo-DFc) electron density map of ATPase domain with ADP complex. The contour level of is 1.0 . **c** (2mFo-DFc) electron density map of ATPase domain with ADP complex. The contour level of is 1.0 .

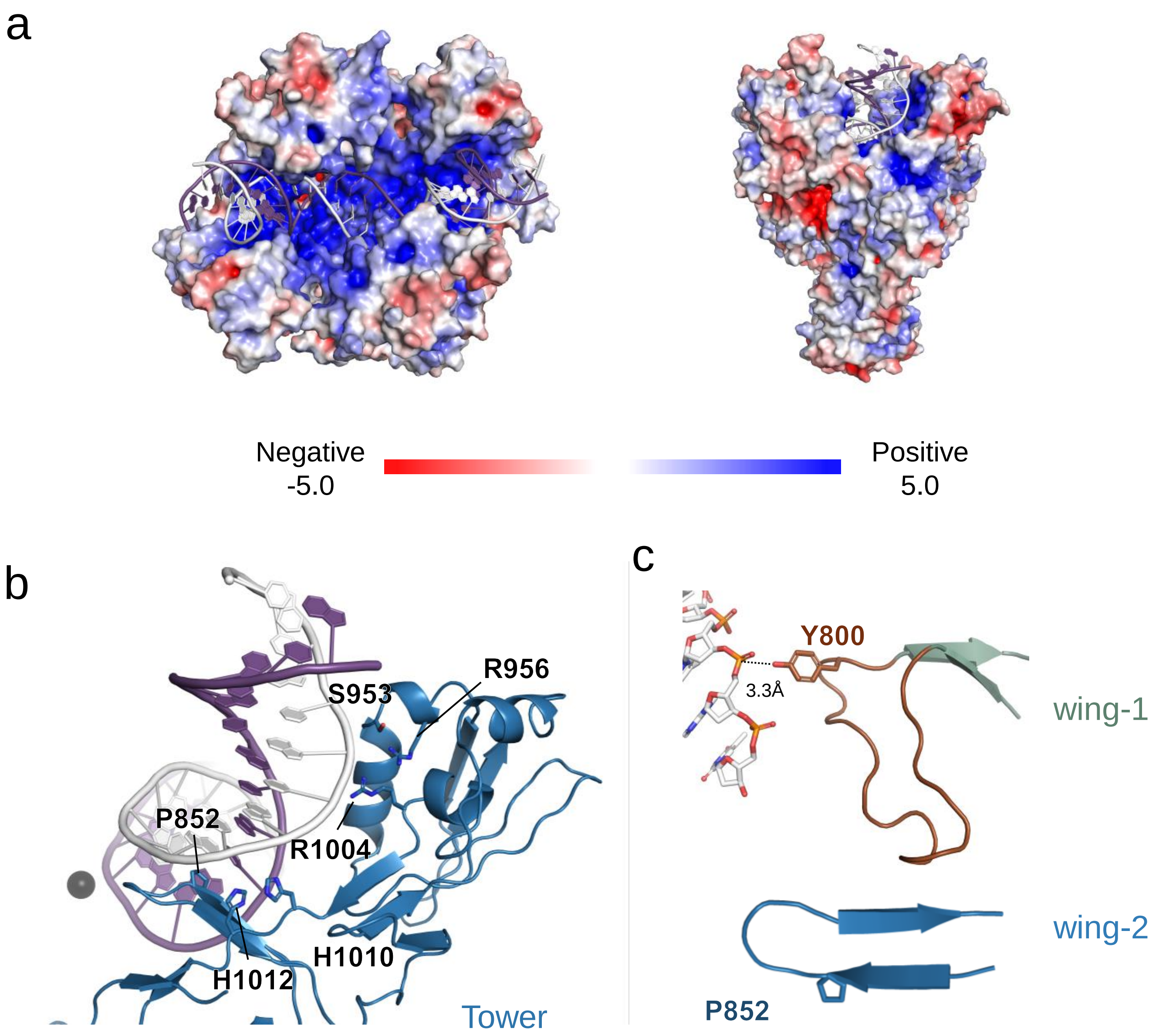

Supplementary Figure 8: Interactions between G-segment DNA and pP1192R. a Electrostatic potential map of the $pP1192R_{CD\text{-}DNA}$ structure from negative (red) to positive (blue), the DNA is shown as cartoon. **b** Magnified view of the interaction interfaces between the Tower subdomain (blue) and DNA (purple). **c** Two conserved β-hairpins within the central domain of pP1192R. Wing-1:β-sheet is colored in green, loop is colored in brown, DNA are shown as sticks with oxygen, nitrogen and Phosphorus atoms shown in red, blue and orange, respectively. Wing-2 is colored in blue.

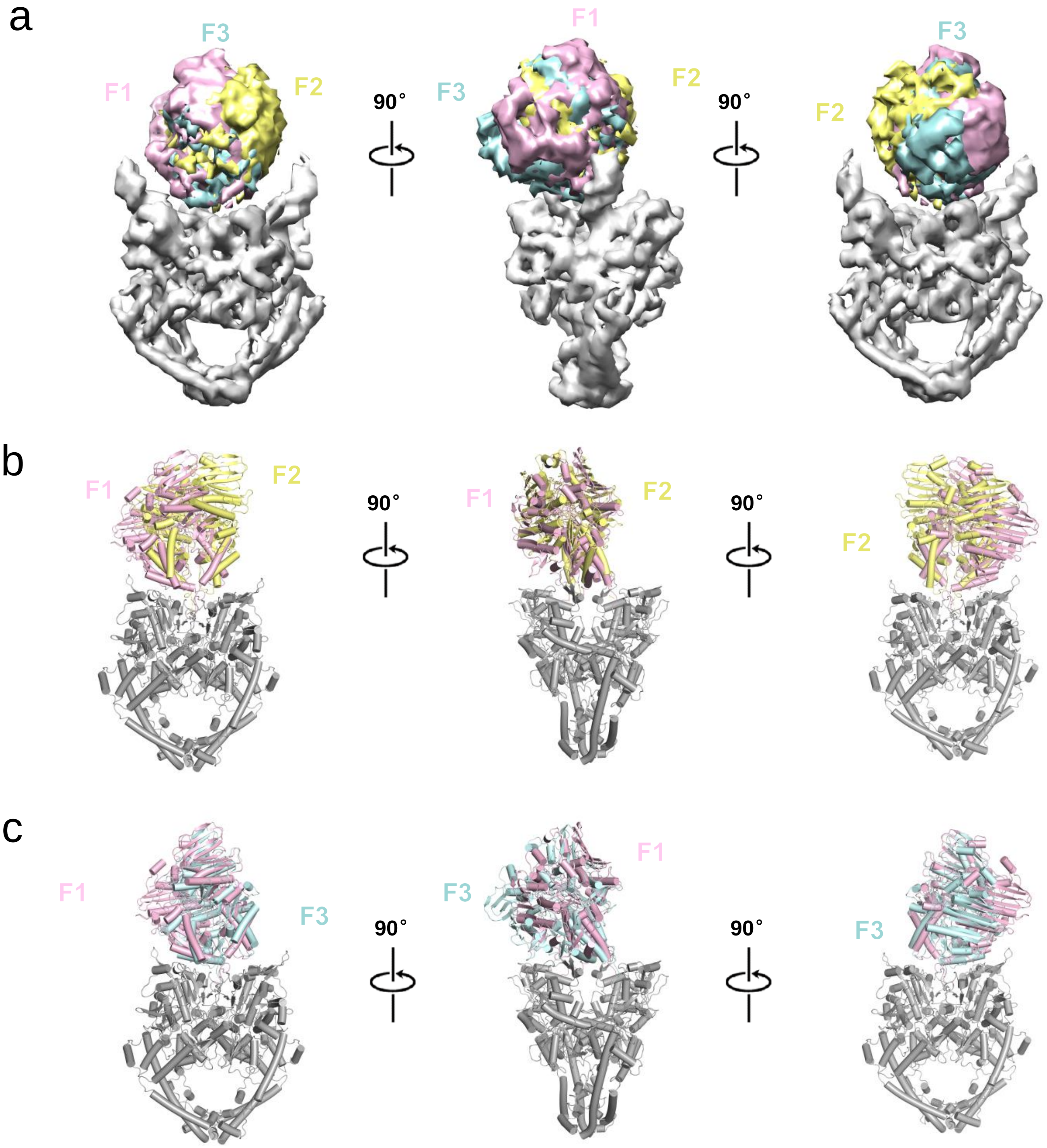

Supplementary Figure 9: Conformational comparison of pP1192R$_{F1\text{-}3}$ ATPase domain.
a Superimposition of pP1192R$_{F1\text{-}3}$ with distinct orientations of the ATPase domain. **b** Superimposition of pP1192R$_{F1}$ and pP1192R$_{F2}$. **c** Superimposition of pP1192R$_{F1}$ and pP1192R$_{F3}$. The ATPase domain is colored by pink (pP1192R$_{F1}$), yellow (pP1192R$_{F2}$), blue (pP1192R$_{F3}$) respectively. The central domain is colored gray.

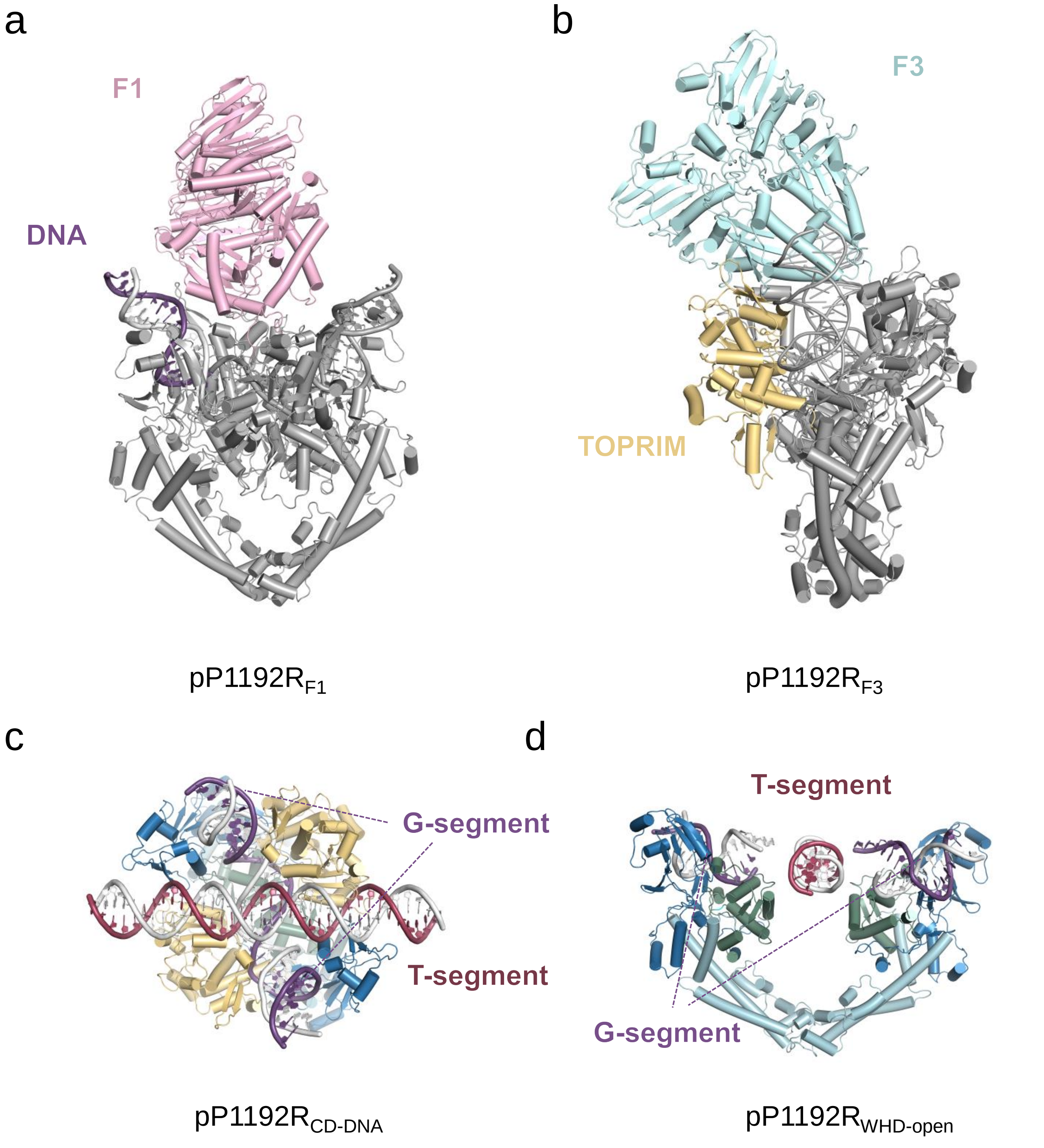

Supplementary Figure 10: Conformational changes of pP1192R and pP1192R-DNA complex.
a The ATPase domain (pink) tilts towards the DNA terminal (purple) in $pP1192R_{F1}$ state. **b** The ATPase domain (blue) tilts towards the TOPRIM subdomain in $pP1192R_{F3}$ state, highlighted in yellow. **c** A modeled T-segment (red) positioned above the G-segment within the $pP1192R_{CD\text{-}DNA}$ structure. G-segment DNA and protein is shown in cartoon and colored as Fig. 2. **d** A modeled T-segment (red) threading through the gap within the structure of $pP1192R_{WHD\text{-}open}$. G-segment DNA and protein is shown in cartoon and colored as Fig. 2.

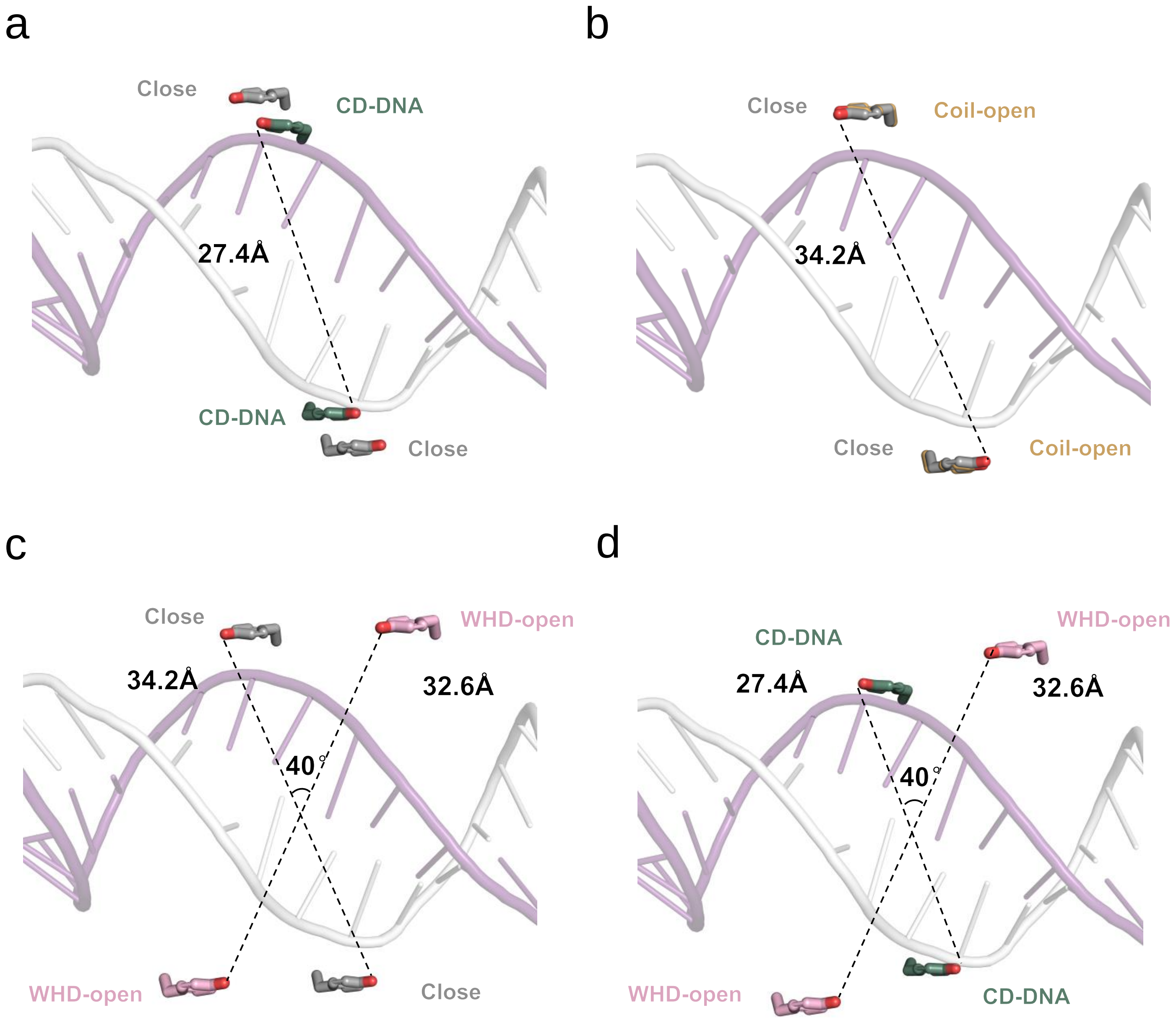

Supplementary Figure 11: The positional variations between two catalytic Y800 in different conformations of pP1192R. Comparison of the positional change between the two Y800 within pP1192R$_{CD\text{-}DNA}$ (green) and pP1192R$_{Close}$ (gray) **(a)**, pP1192R$_{Coil\text{-}open}$ (orange) and pP1192R$_{Close}$ **(b)**, pP1192R$_{WHD\text{-}open}$ (pink) and pP1192R$_{Close}$ **(c)**, pP1192R$_{CD\text{-}DNA}$ and pP1192R$_{WHD\text{-}open}$ **(d)**, respectively. DNA is shown in surface and colored as Fig. 2.

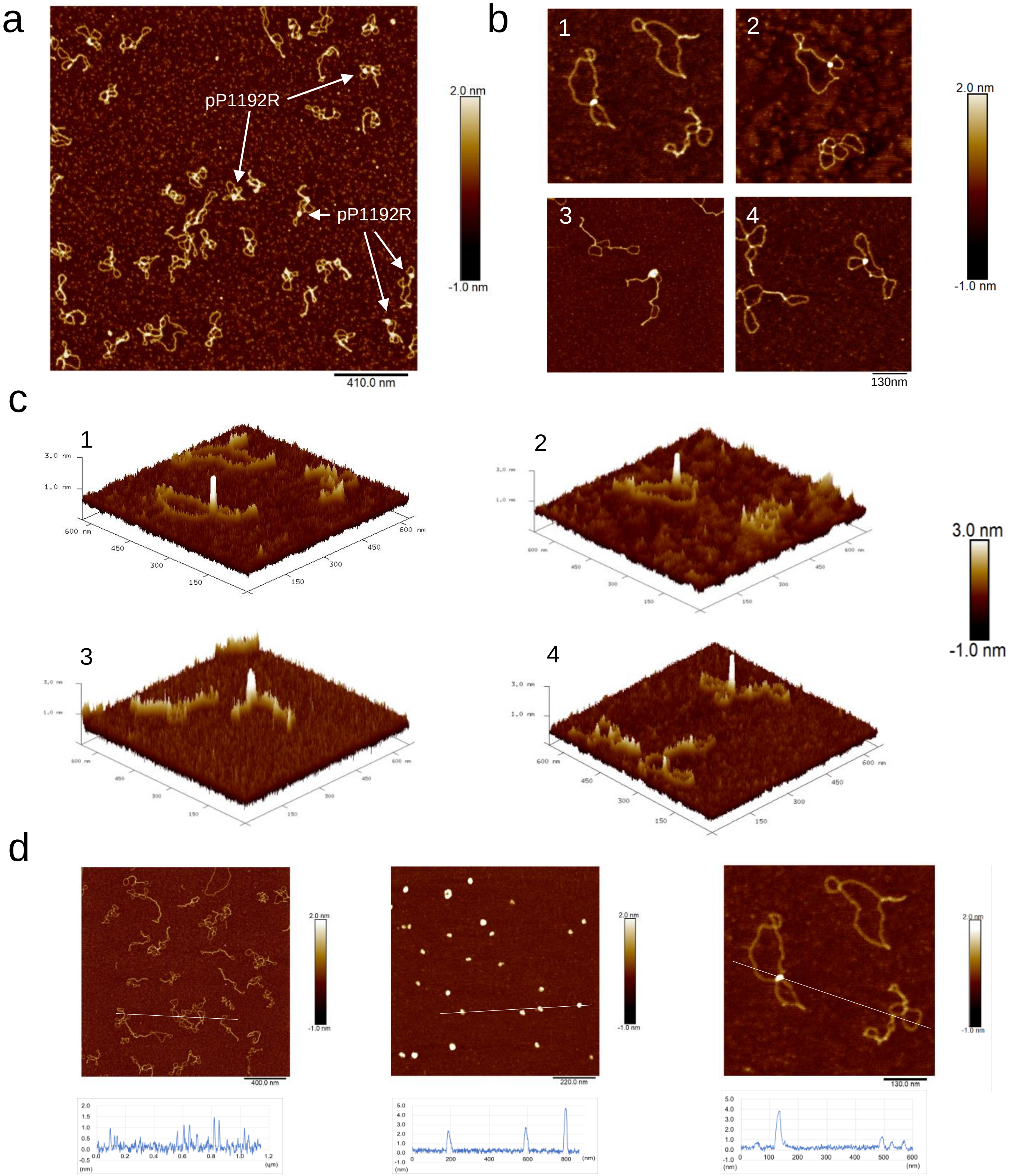

Supplementary Figure 12: Interaction of pP1192R with DNA crossovers. a Representative AFM image of pP1192R binding to crossover of negatively supercoiled plasmid pUC19 (Arrows indicate pP1192R binding to the DNA crossovers) . **b** Close-up view of the interaction between pP1192R and plasmid crossover. **c** 3D AFM image of **b**. **d** Representative AFM image of negatively supercoiled plasmid pUC19 (left), pP1192R (middle), and pP1192R bound to crossover site (right), respectively. Cross sections were displayed. Source data are provided as a Source Data file.

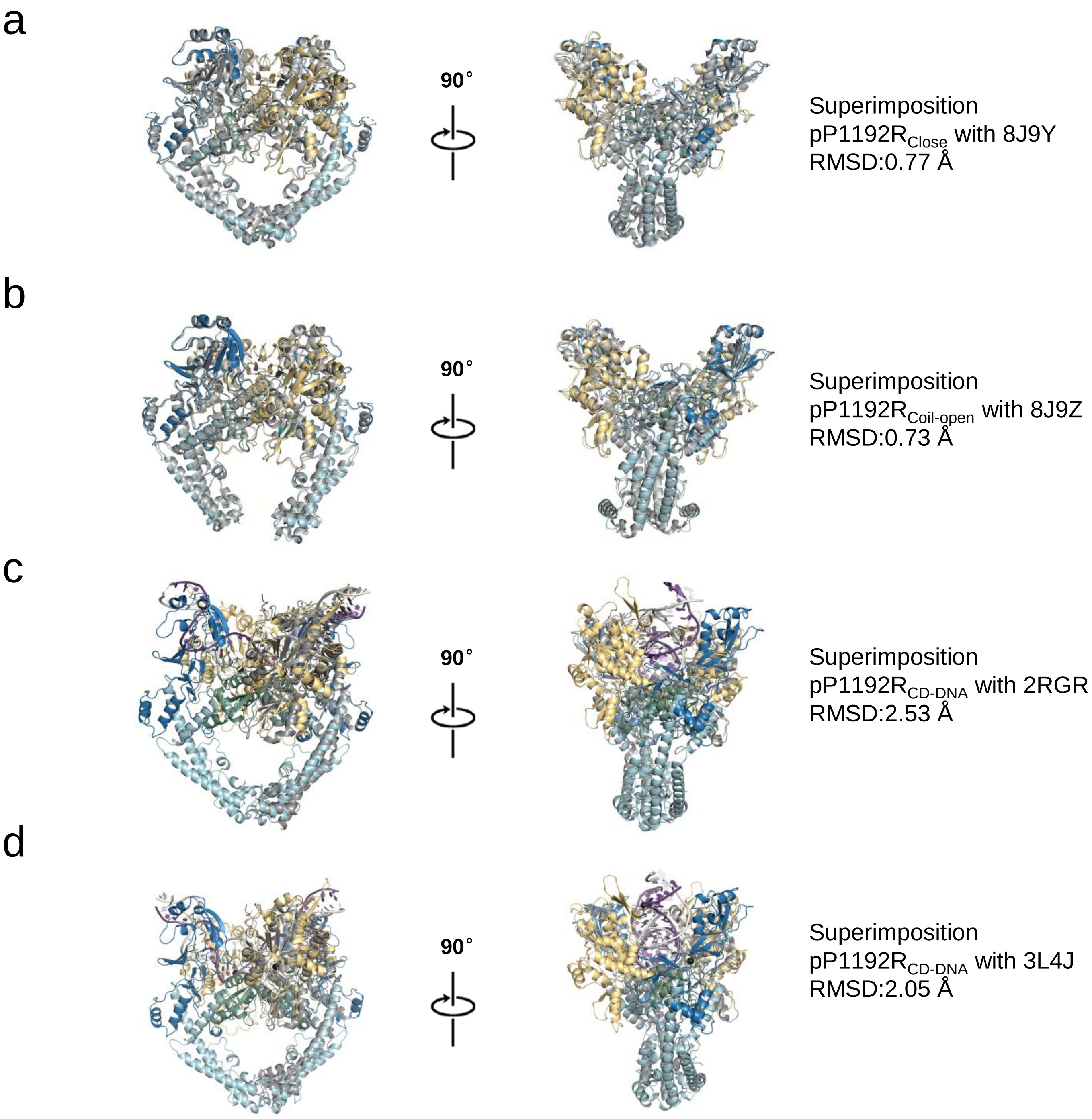

Supplementary Figure 13: The structure comparison of pP1192R in this work with other published model. The model of this work is in the same color as Fig.2b. The published other models is colored in gray. **a** The superimposition of the pP1192R$_{Close}$ with 8J9Y (ASFV) with an RMSD value of 0.77 Å. **b** The superimposition of the pP1192R$_{Coil\text{-}open}$ with 8J9Z (ASFV) with an RMSD value of 0.73 Å. **c** The superimposition of the pP1192RCD-DNA with 2RGR (*S. cerevisiae*) with an RMSD value of 2.53 Å. d The superimposition of the pP1192RCD-DNA with 3L4J (*S. cerevisiae*) with an RMSD value of 2.05 Å. DNA and protein is shown in cartoon and colored as Fig. 2.

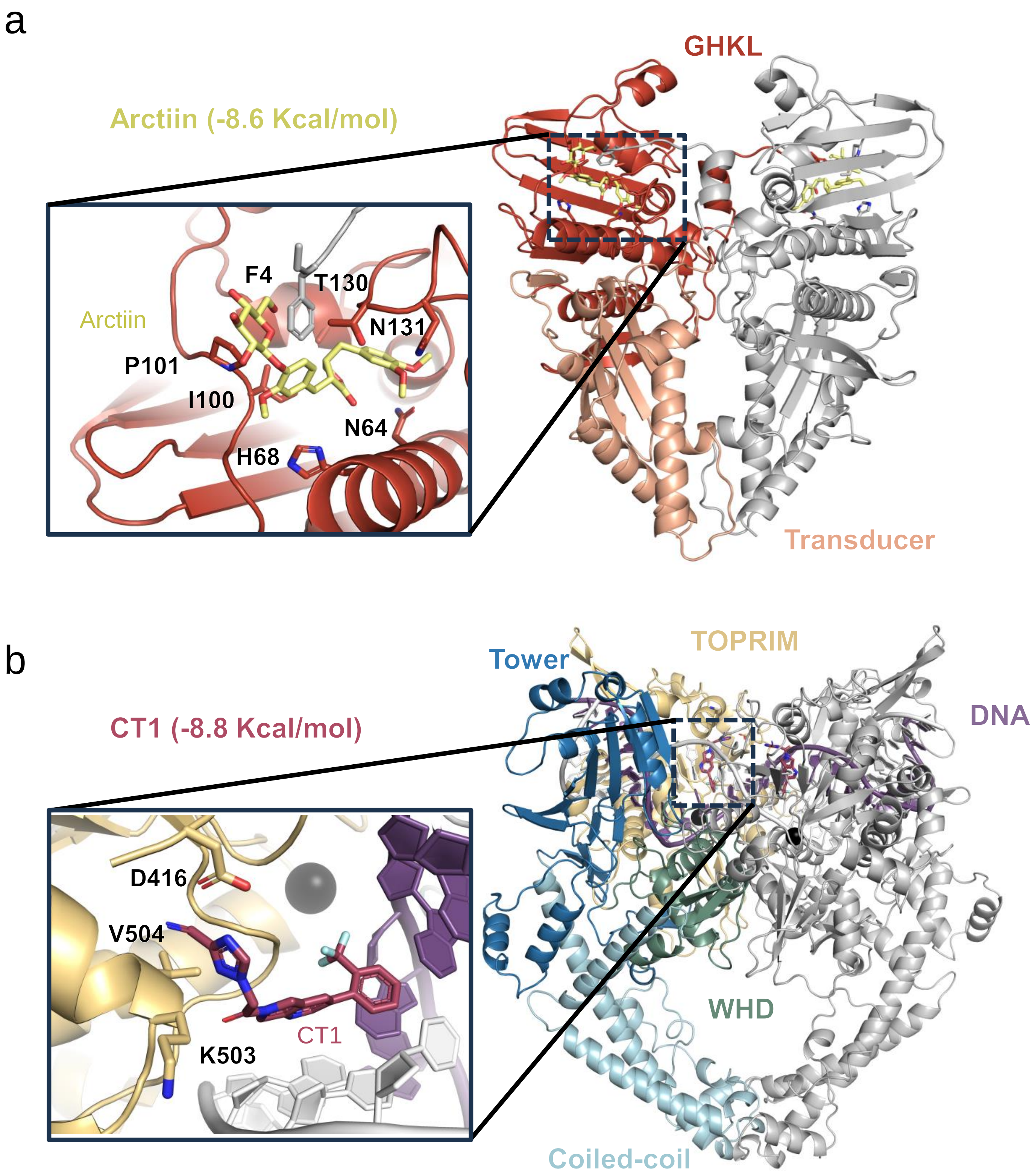

Supplementary Figure 14: Molecular docking analysis of pP1192R. a Docking Arctiin (yellow) into the crystal structures of ATPase domain complexed with AMPPNP. Prior to Autodock, the AMPPNP was removed. The binding affinity of arctiin to ATPase domain is -8.6 Kcal/mol. **b** Docking CT1 (from PDB 8GCC) into structure of pP1192R$_{CD\text{-}DNA}$. The binding affinity of CT1 (Magenta) to pP1192R$_{CD\text{-}DNA}$ is -8.8 Kcal/mol. DNA and protein is shown in cartoon and colored as Fig. 2.

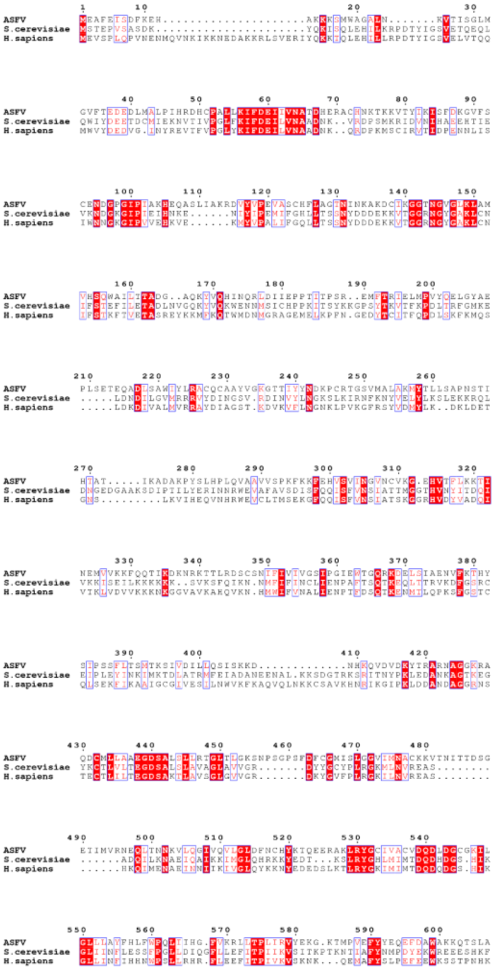

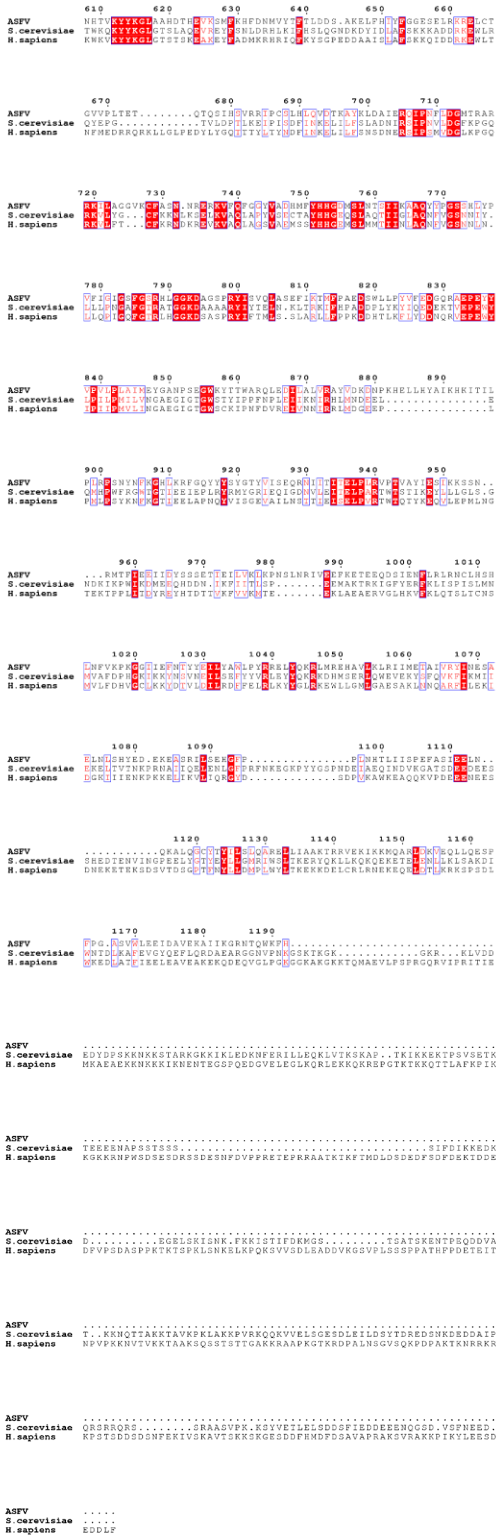

Supplementary Figure 15: Sequence alignment of pP1192R with Topo II of *S. cerevisiae* and *H. sapiens*. The percentage of identity between pP1192R and Topo II of *S. cerevisiae* is 26.05%. The percentage of identity between pP1192R and Topo II of *H. sapiens* is 25.88%.

Supplementary Table 1: Statistics of Data Collection, Image Processing and Model Building of pP1192R-DNA complex.

	CD-DNA	F1	F2	F3
**Data collection**				
**EM equipment**	FEI Titan Krios	FEI Titan Krios	FEI Titan Krios	FEI Titan Krios
**Voltage (kV)**	300	300	300	300
**Detector**	Gatan K2	Gatan K2	Gatan K2	Gatan K2
**Pixel size (Å/pixel)**	0.65	0.82	0.82	0.82
**Electron dose (e^-/Å2)**	60	60	60	60
**Defocus range (μm)**	-1.2-2.5	-1.2-2.5	-1.2-2.5	-1.2-2.5
**Reconstruction**				
**Software**	Relion3.0	Relion3.0	Relion3.0	Relion3.0
**Number of used particles**	102k	19k	19k	15k
**Symmetry**	C1	C1	C1	C1
**Map sharpening B-factor (Å^2)**	-106	-80	-80	-80
**Final resolution (Å)**	3.2	5.6	4.8	5.9
**Model building**				
**Software**	Coot	Coot	Coot	Coot
**Model Refinement**				
**Software**	PHENIX	PHENIX	PHENIX	PHENIX
**Map CC (mask)**	0.836	0.608	0.459	0.578
**Map CC (peaks)**	0.712	0.517	0.415	0.482
**Map CC (volume)**	0.811	0.597	0.482	0.562
**Rmsd (bonds) (Å)**	0.0084	0.0088	0.0088	0.0088
**Rmsd (angles) (º)**	1.21	1.24	1.24	1.25
**Validation**				
**MolProbity score**	1.49	1.99	1.84	1.86
**Clash score**	3.91	13.87	11.80	11.79
**Ramachandran plot**				
**Outliers (%)**	0.0	0.0	0.0	0.0
**Allowed (%)**	4.1	3.5	3.2	3.2
**Favored (%)**	95.9	96.5	96.8	96.8
**Rotamer outliers (%)**	0.15	1.15	0.73	0.15
**Cβ outliers (%)**	0	0	0	0

Supplementary Table 2: Statistics of Data Collection, Image Processing and Model Building of pP1192R.

	Coil-open	Close	WHD-open
**Data collection**			
**EM equipment**	FEI Titan Krios	FEI Titan Krios	FEI Titan Krios
**Voltage (kV)**	300	300	300
**Detector**	Gatan K2	Gatan K2	Gatan K2
**Pixel size (Å/pixel)**	0.65	0.65	0.65
**Electron dose (e^-/Å2)**	60	60	60
**Defocus range (μm)**	-1.2-2.5	-1.2-2.5	-1.2-2.5
**Reconstruction**			
**Software**	Relion3.0	Relion3.0	Relion3.0
**Number of used particles**	150k	102k	48k
**Symmetry**	C2	C2	C2
**Map sharpening B-factor (Å^2)**	-148	-160	-201
**Final resolution (Å)**	3.3	3.4	4.3
**Model building**			
**Software**	Coot	Coot	Coot
**Model Refinement**			
**Software**	PHENIX	PHENIX	PHENIX
**Map CC (mask)**	0.848	0.825	0.769
**Map CC (peaks)**	0.689	0.654	0.679
**Map CC (volume)**	0.824	0.789	0.758
**Rmsd (bonds) (Å)**	0.0110	0.0109	0.0076
**Rmsd (angles) (º)**	1.31	1.40	1.25
**Validation**			
**MolProbity score**	1.66	1.53	1.92
**Clash score**	4.97	2.80	8.04
**Ramachandran plot**			
**Outliers (%)**	0.0	0.0	0.0
**Allowed (%)**	4.9	6.1	5.6
**Favored (%)**	95.1	93.9	94.4
**Rotamer outliers (%)**	0.76	0.54	0.00
**Cβ outliers (%)**	0	0	0

Supplementary Table 3: Statistics for Data Collection and refinement of ATPase domain structures

	ATPase-AMPPNP	ATPase-ADP
**Data collection**		
**Space group**	$P6_122$	$P6_122$
**Cell dimensions**		
**a, b, c (Å)**	85.08, 85.08, 210.18	85.23, 85.23, 209.81
**α, β, γ (°)**	90, 90, 120	90, 90, 120
**Resolution (Å)**	50.00-2.60 (2.64-2.60)[a]	50.00-2.30 (2.34-2.30)
**R_{merge}**	0.203 (1.019)	0.149 (0.945)
**I/σI**	11.00 (1.38)	14.22 (2.24)
**Completeness (%)**	100.0 (99.3)	99.4 (99.4)
**Redundancy**	19.0 (9.0)	8.2 (7.7)
**No. of reflections**	277,001	170,228
**No. of unique reflections**	14,604	20,799
**Refinement**		
**R_{work}/ R_{free}**	0.200/0.244	0.193/0.246
**No. of atoms**		
**Protein**	3,141	3,080
**Ligand/ion**	32	27
**Water**	121	108
**Average B-factors**		
**Protein**	37.0	43.7
**Ligand/ion**	24.7	34.1
**Water**	31.9	38.1
**R.m.s deviations**		
**Bond lengths (Å)**	0.010	0.004
**Bond angles (°)**	1.060	1.135
**Ramachandran plot (%)**		
**Favoured**	98.5	98.5
**Allowed**	1.5	1.2
**Outliers**	0	0.3

[a]Values in parentheses correspond to the shell of the highest resolution

Supplementary Table 4: Summary of the models

Subunit Name	Chain	Total residues/ range built	Unmodelled residues	% atomic model
		**$pP1192R_{CD-DNA}$**		
pP1192R	A	1192/413-1192	1-412	780/1192
pP1192R	B	1192/415-1192	1-414	778/1192
DNA	C	52/17-48	1-16, 49-52	32/52
DNA	D	52/5-37	1-4, 38-52	33/52
		**$pP1192R_{F1}$**		
pP1192R	A	1192/3-1192	1-2	1190/1192
pP1192R	B	1192/3-1192	1-2	1190/1192
DNA	C	52/14-51	1-13, 52	38/52
DNA	D	52/4-41	1-3, 42-52	38/52
		**$pP1192R_{F2}$**		
pP1192R	A	1192/3-403, 413-1192	1-2, 404-412	1181/1192
pP1192R	B	1192/3-410, 415-1192	1-2, 411-414	1186/1192
DNA	C	52/14-51	1-13, 52	38/52
DNA	D	52/4-41	1-3, 42-52	38/52
		**$pP1192R_{F3}$**		
pP1192R	A	1192/3-1192	1-2	1190/1192
pP1192R	B	1192/3-405, 416-1192	1-2, 406-415	1180/1192
DNA	C	52/14-51	1-13, 52	38/52
DNA	D	52/4-41	1-3, 42-52	38/52

Supplementary Table 4: Summary of the models

Subunit Name	Chain	Total residues/ range built	Unmodelled residues	% atomic model
**pP1192R$_{\text{Coil-open}}$**				
pP1192R	A	1192/415-472, 501-893, 895-1192	1-414, 473-500, 894	749/1192
pP1192R	B	1192/415-472, 501-893, 895-1192	1-414, 473-500, 894	749/1192
**pP1192R$_{\text{Close}}$**				
pP1192R	A	1192/415-473, 502-852, 856-893, 895-1192	1-414, 474-501, 853-855, 894	746/1192
pP1192R	B	1192/415-473, 502-852, 856-893, 895-1192	1-414, 474-501, 853-855, 894	746/1192
**pP1192R$_{\text{WHD-open}}$**				
pP1192R	A	1192/701-1192	1-700	492/1192
pP1192R	B	1192/701-1192	1-700	/1492192
**ATPase-AMPPNP**				
pP1192R	A	434/3-405	1-2, 406-434	403/434
**ATPase-ADP**				
pP1192R	A	434/3-334, 342-405	1-2, 335-341	396/434

Supplementary Table 5: DNA oligonucleotides

52bp dsDNA
5’-ATGCATATATATGTATATGTATGTGTGTATATAT ACACATATATATATATAT-3’
5’-ATATATATATATATGTGTATATATACACACATAC ATATACATATATATGCAT-3’

Four-way junction DNA
O1: 5’-CTGGACGCAATCTGACAATGCGCTCATCGTC ATCCTCGGCACGCGCCG-3’
O2: 5’-CGGCGCGTGCCGAGGATGACGATGAGATAG GCGTTAACGCGGCCTA-3’
O3: 5’-TAGGCCGCGTTAACGCCTATTTGCCCGGGAG TACCGGCATTCCT-3’
O4: 5’-AGGAATGCCGGTACTCCCGGGCAACGCATTG TCAGATTGCGTCCAG-3’
