# Supplementary material for: Structural insights into the DNA topoisomerase II of the African swine fever virus
Source: Nat Commun. 2024 May 30;15:4607. doi: 10.1038/s41467-024-49047-w (PMC11139879; doi:10.1038/s41467-024-49047-w)
Supplement: Supplementary file 3 — Reporting Summary [file 41467_2024_49047_MOESM3_ESM.pdf]

nature portfolio

Corresponding author(s): Xuemei Li, Zihe Rao, Yutao Chen

Last updated by author(s): Apr 15, 2024

Reporting Summary

Nature Portfolio wishes to improve the reproducibility of the work that we publish. This form provides structure for consistency and transparency in reporting. For further information on Nature Portfolio policies, see our Editorial Policies and the Editorial Policy Checklist.

Statistics

For all statistical analyses, confirm that the following items are present in the figure legend, table legend, main text, or Methods section.

n/a	Confirmed	
☐	☒	The exact sample size (*n*) for each experimental group/condition, given as a discrete number and unit of measurement
☐	☒	A statement on whether measurements were taken from distinct samples or whether the same sample was measured repeatedly
☒	☐	The statistical test(s) used AND whether they are one- or two-sided<br>*Only common tests should be described solely by name; describe more complex techniques in the Methods section.*
☒	☐	A description of all covariates tested
☒	☐	A description of any assumptions or corrections, such as tests of normality and adjustment for multiple comparisons
☐	☒	A full description of the statistical parameters including central tendency (e.g. means) or other basic estimates (e.g. regression coefficient) AND variation (e.g. standard deviation) or associated estimates of uncertainty (e.g. confidence intervals)
☒	☐	For null hypothesis testing, the test statistic (e.g. *F*, *t*, *r*) with confidence intervals, effect sizes, degrees of freedom and *P* value noted<br>*Give P values as exact values whenever suitable.*
☒	☐	For Bayesian analysis, information on the choice of priors and Markov chain Monte Carlo settings
☒	☐	For hierarchical and complex designs, identification of the appropriate level for tests and full reporting of outcomes
☒	☐	Estimates of effect sizes (e.g. Cohen's *d*, Pearson's *r*), indicating how they were calculated

Our web collection on statistics for biologists contains articles on many of the points above.

Software and code

Policy information about availability of computer code

Data collection	eSerialEM 4-0-9
Data analysis	Open Babel 2.4.1, AutoDock vina 1.1.2, relion 3.0.8, UCSF Chimera 1.15, PyMoL 4.6.0, Clustal X 1.3, ESPript 3.0, Coot 0.9.2, PHENIX 1.18.2

For manuscripts utilizing custom algorithms or software that are central to the research but not yet described in published literature, software must be made available to editors and reviewers. We strongly encourage code deposition in a community repository (e.g. GitHub). See the Nature Portfolio guidelines for submitting code & software for further information.

Data

Policy information about availability of data

All manuscripts must include a data availability statement. This statement should provide the following information, where applicable:

- Accession codes, unique identifiers, or web links for publicly available datasets
- A description of any restrictions on data availability
- For clinical datasets or third party data, please ensure that the statement adheres to our policy

The structures of pP1192R and its complexes have been deposited at the Protein Data Bank (PDB). The accession codes are: 8KGR [https://doi.org/10.2210/pdb8KGR/pdb] (pP1192RCD-DNA), 8KGO [https://doi.org/10.2210/pdb8KGO/pdb] (pP1192RClose), 8KGP [https://doi.org/10.2210/pdb8KGP/pdb] (pP1192RCoil-open), 8KGL [https://doi.org/10.2210/pdb8KGL/pdb] (pP1192RWHD-open), 8KGQ [https://doi.org/10.2210/pdb8KGQ/pdb] (pP1192RF1), 8KGM [https://doi.org/10.2210/pdb8KGM/pdb] (pP1192RF2), 8KGN [https://doi.org/10.2210/pdb8KGN/pdb] (pP1192RF3), 8KGT [https://doi.org/10.2210/pdb8KGT/pdb] (ATPase-

ADP) and 8KGS [https://doi.org/10.2210/pdb8KGS/pdb] (ATPase-AMPPNP). The cryo-EM density maps of pP1192R and its complexes have been deposited at the Electron Microscopy Data Bank. The accession codes are: EMD-37231 [https://www.ebi.ac.uk/pdbe/entry/emdb/EMD-37231] (pP1192RCD-DNA), EMD-37228 [https://www.ebi.ac.uk/pdbe/entry/emdb/EMD-37228] (pP1192RClose), EMD-37229 [https://www.ebi.ac.uk/pdbe/entry/emdb/EMD-37229] (pP1192RCoil-open), EMD-37225 [https://www.ebi.ac.uk/pdbe/entry/emdb/EMD-37225] (pP1192RWHD-open), EMD-37230 [https://www.ebi.ac.uk/pdbe/entry/emdb/EMD-37230] (pP1192RF1), EMD-37226 [https://www.ebi.ac.uk/pdbe/entry/emdb/EMD-37226] (pP1192RF2) and EMD-37227 [https://www.ebi.ac.uk/pdbe/entry/emdb/EMD-37227] (pP1192RF3). The previously available coordinates we used for structural analysis include 2RGR [https://doi.org/10.2210/pdb2RGR/pdb] (step2 in Figure 7), 3L4J [https://doi.org/10.2210/pdb3L4J/pdb] (step4 in Figure 7) and 8GCC [https://doi.org/10.2210/pdb8GCC/pdb] (CT1). The SDF structure files for Arctiin (https://pubchem.ncbi.nlm.nih.gov/compound/100528) were obtained from the PubChem database. AlphaFold models used for model building are included in the Source Data file. Additional AFM images have been uploaded to Figshare (https://doi.org/10.6084/m9.figshare.25273231.v1). Source data are provided with this paper.

Research involving human participants, their data, or biological material

Policy information about studies with human participants or human data. See also policy information about sex, gender (identity/presentation), and sexual orientation and race, ethnicity and racism.

Reporting on sex and gender	N/A
Reporting on race, ethnicity, or other socially relevant groupings	N/A
Population characteristics	N/A
Recruitment	N/A
Ethics oversight	N/A

Note that full information on the approval of the study protocol must also be provided in the manuscript.

Field-specific reporting

Please select the one below that is the best fit for your research. If you are not sure, read the appropriate sections before making your selection.

☒ Life sciences ☐ Behavioural & social sciences ☐ Ecological, evolutionary & environmental sciences

For a reference copy of the document with all sections, see nature.com/documents/nr-reporting-summary-flat.pdf

Life sciences study design

All studies must disclose on these points even when the disclosure is negative.

Sample size	For the cryo-EM analysis, the number of micrographys is determined by the available microscope time. The sample size of ATPase assay was performed three times, which is sufficient for statistic analysis.
Data exclusions	No data were excluded from analyses.
Replication	Sample preparation-related experiments including protein purification and enzymatic assays were reproduced at least three times independently. All attempts at replication were successful.
Randomization	Randomization is not relevant to cryo-EM and other experiments, because the sample were not allocated into experimental groups during data acqyisition and analysis.
Blinding	Blinding is not relevant to this study. The parameters for biochemistry, cryo-EM, and any other experiments in this study did not require subjective assessments of the treatments.

Reporting for specific materials, systems and methods

We require information from authors about some types of materials, experimental systems and methods used in many studies. Here, indicate whether each material, system or method listed is relevant to your study. If you are not sure if a list item applies to your research, read the appropriate section before selecting a response.

Materials & experimental systems

n/a	Involved in the study
☒	☐ Antibodies
☐	☒ Eukaryotic cell lines
☒	☐ Palaeontology and archaeology
☒	☐ Animals and other organisms
☒	☐ Clinical data
☒	☐ Dual use research of concern
☒	☐ Plants

Methods

n/a	Involved in the study
☒	☐ ChIP-seq
☒	☐ Flow cytometry
☒	☐ MRI-based neuroimaging

Eukaryotic cell lines

Policy information about cell lines and Sex and Gender in Research

Cell line source(s)	Spodoptera frugiperda, Sf9, purchased from Invitrogen (ThermoFisher).
Authentication	Not authenticated.
Mycoplasma contamination	Sf9 cell line tested negative for mycoplamsa contamination.
Commonly misidentified lines (See ICLAC register)	No commonly misidentified cell lines were used in the study.

Plants

Seed stocks	N/A
Novel plant genotypes	N/A
Authentication	N/A
