# Supplementary figures and images for: Structural insights into the DNA topoisomerase II of the African swine fever virus
Source: Nat Commun. 2024 May 30;15:4607. doi: 10.1038/s41467-024-49047-w (PMC11139879; doi:10.1038/s41467-024-49047-w)

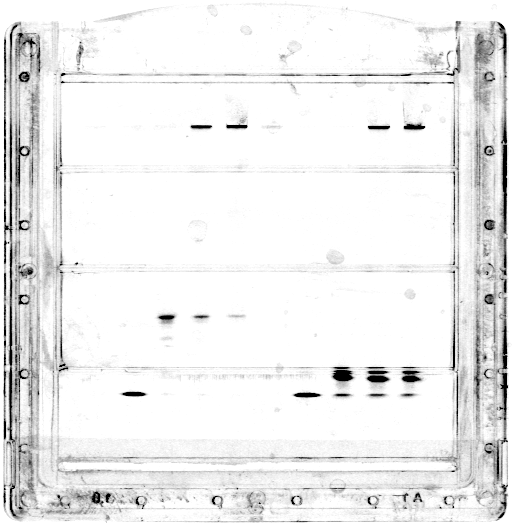

Supplement: Supplementary file 4 — Source data [file 41467_2024_49047_MOESM4_ESM.zip › Source Data/Fig 6a.PNG]

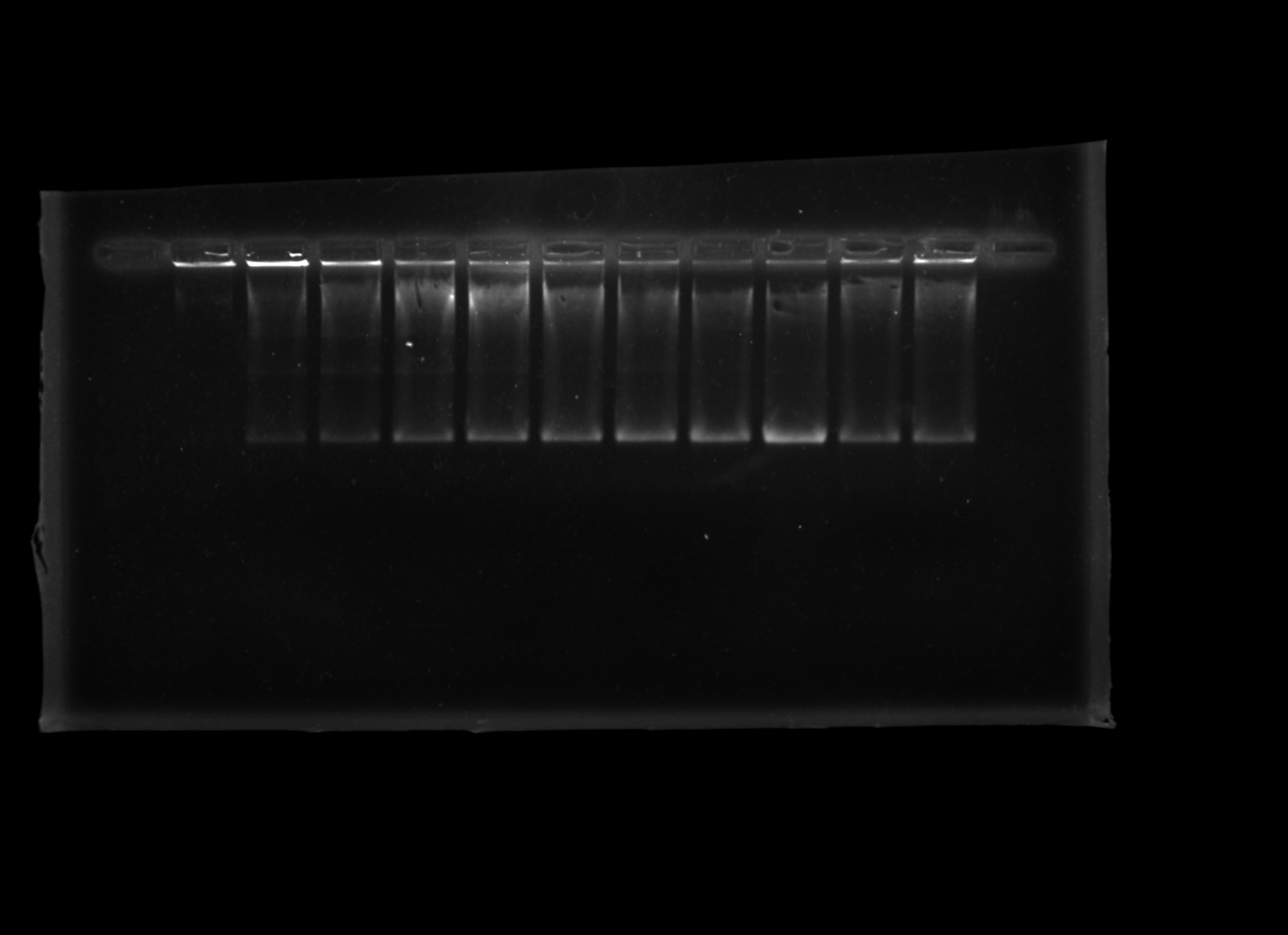

Supplement: Supplementary file 4 — Source data [file 41467_2024_49047_MOESM4_ESM.zip › Source Data/Fig1a bottom left.tif]

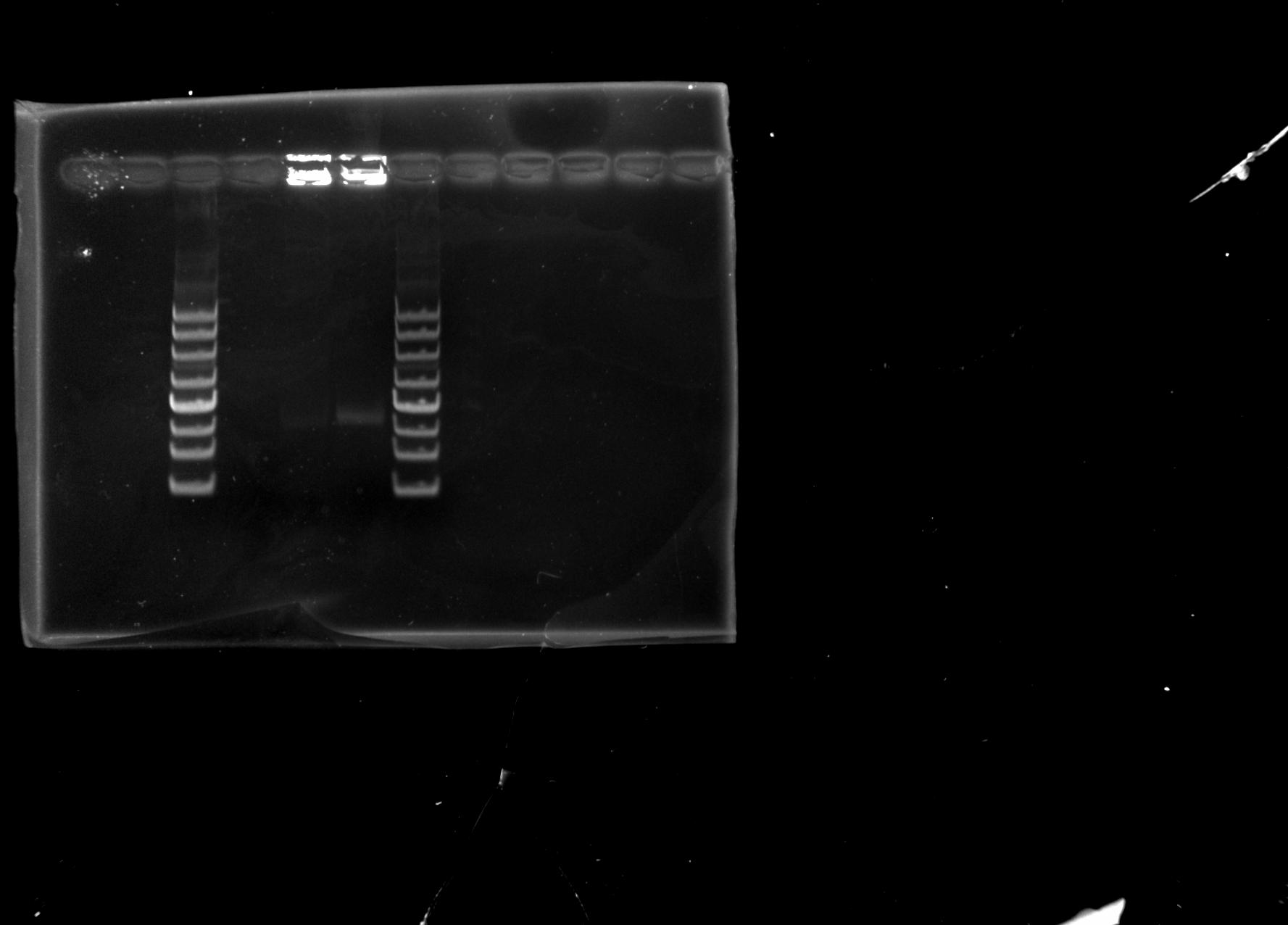

Supplement: Supplementary file 4 — Source data [file 41467_2024_49047_MOESM4_ESM.zip › Source Data/Fig1a bottom right.tif]

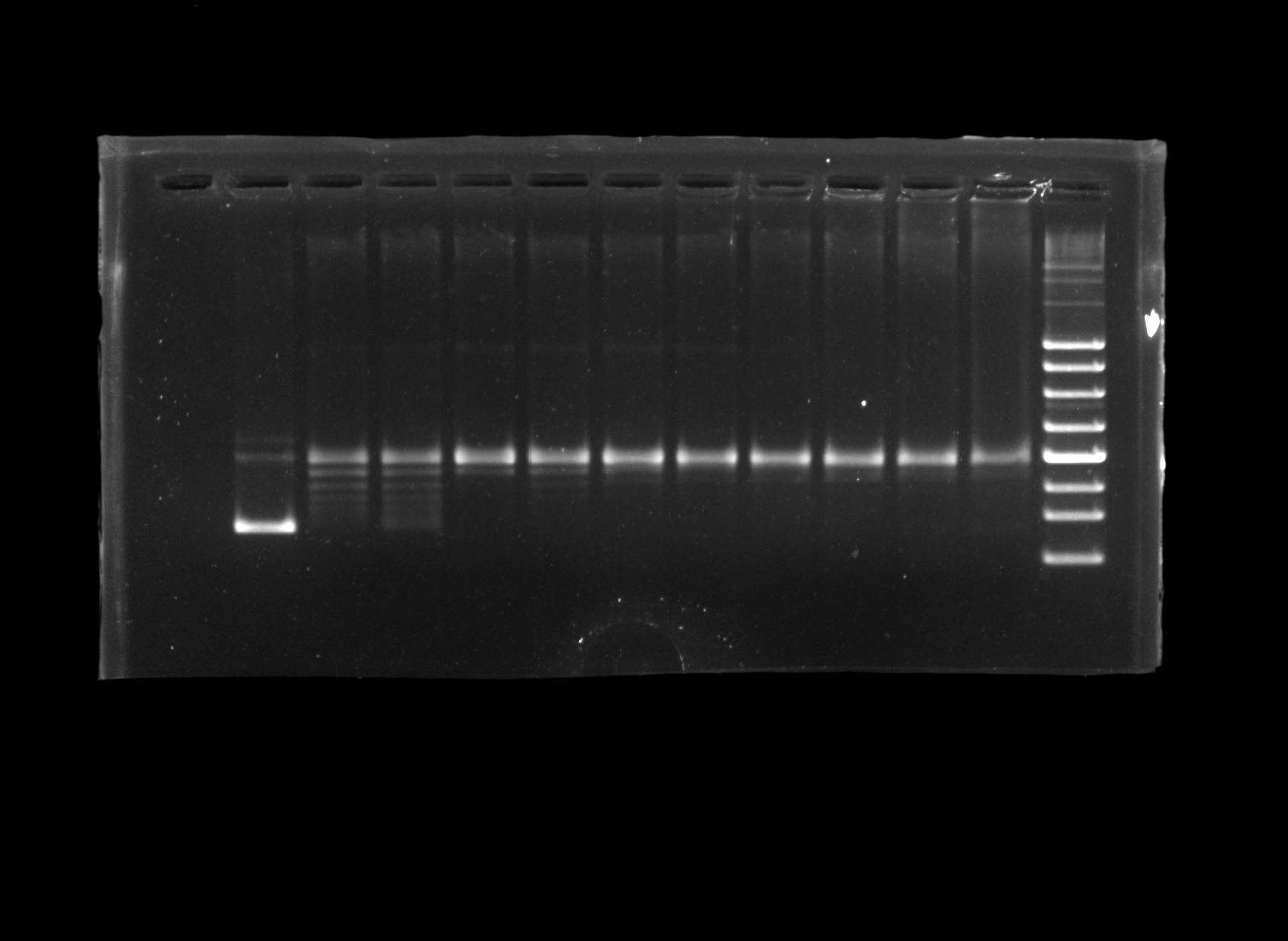

Supplement: Supplementary file 4 — Source data [file 41467_2024_49047_MOESM4_ESM.zip › Source Data/Fig1a top.tif]

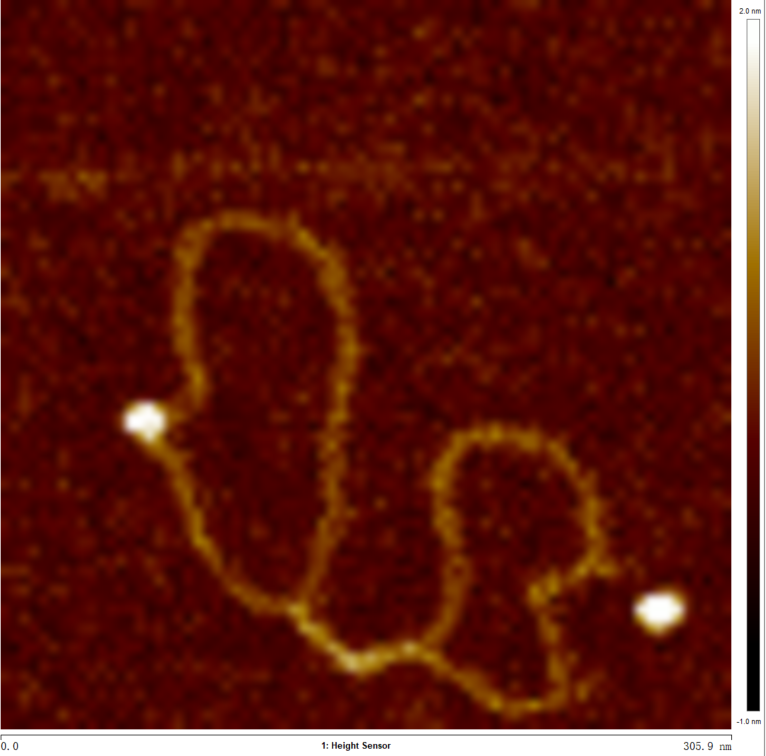

Supplement: Supplementary file 4 — Source data [file 41467_2024_49047_MOESM4_ESM.zip › Source Data/Rseponses to reviewers/1705906008781.png]

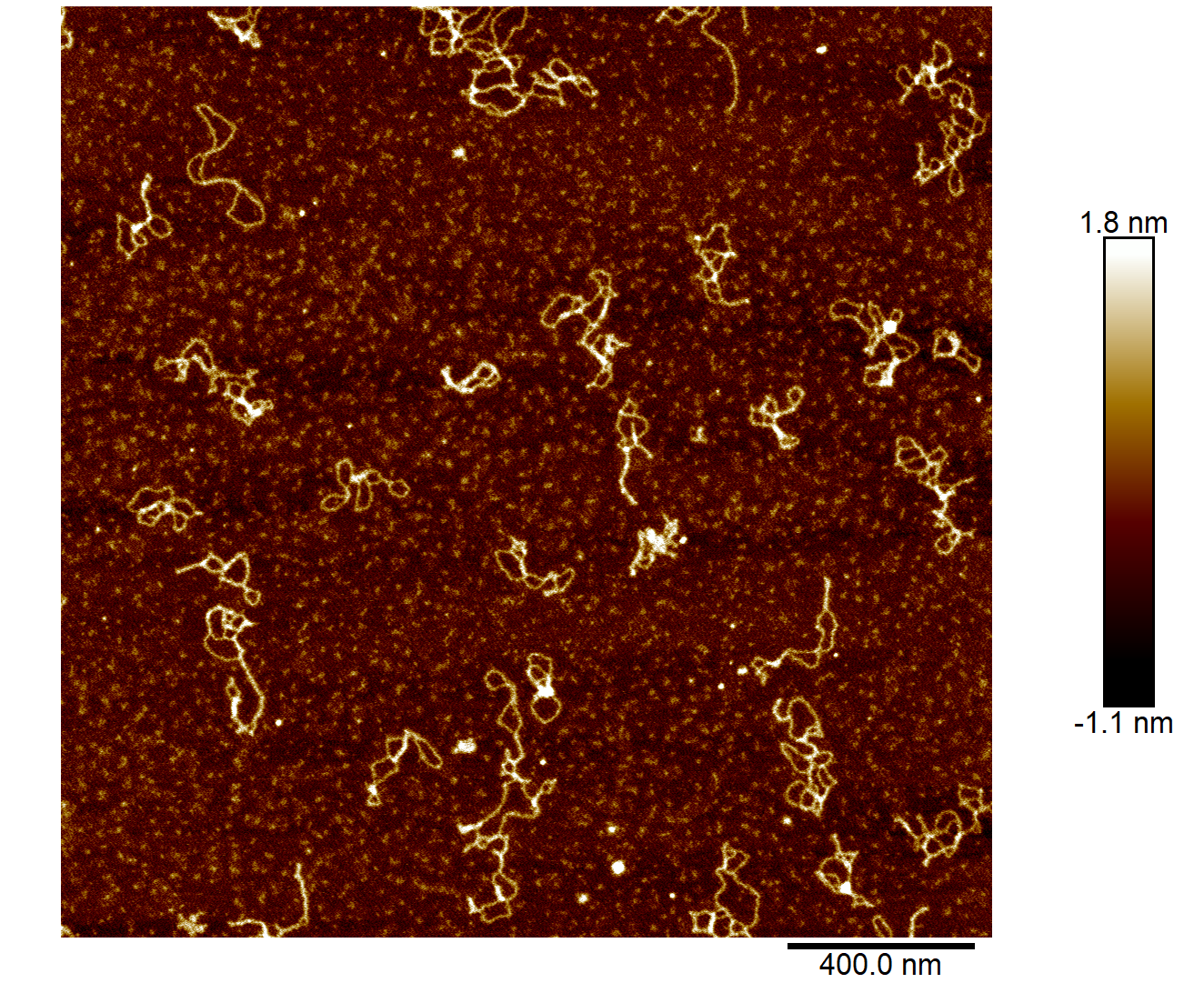

Supplement: Supplementary file 4 — Source data [file 41467_2024_49047_MOESM4_ESM.zip › Source Data/Rseponses to reviewers/20-5-1-1_1.spm.tif]

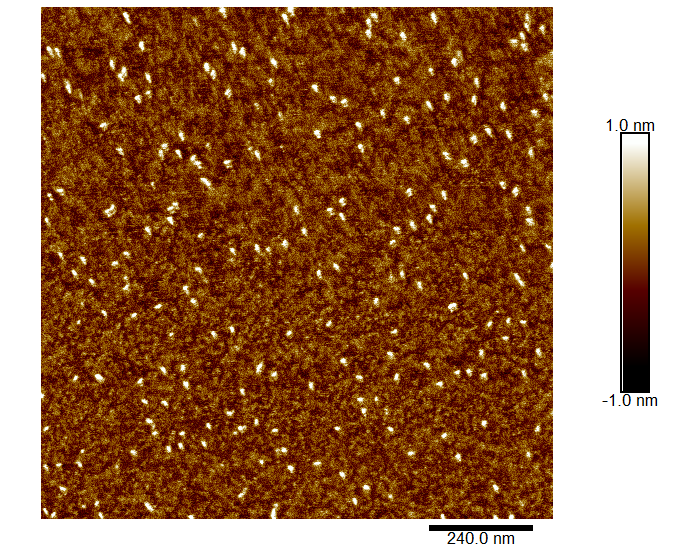

Supplement: Supplementary file 4 — Source data [file 41467_2024_49047_MOESM4_ESM.zip › Source Data/Rseponses to reviewers/four_way.tif]

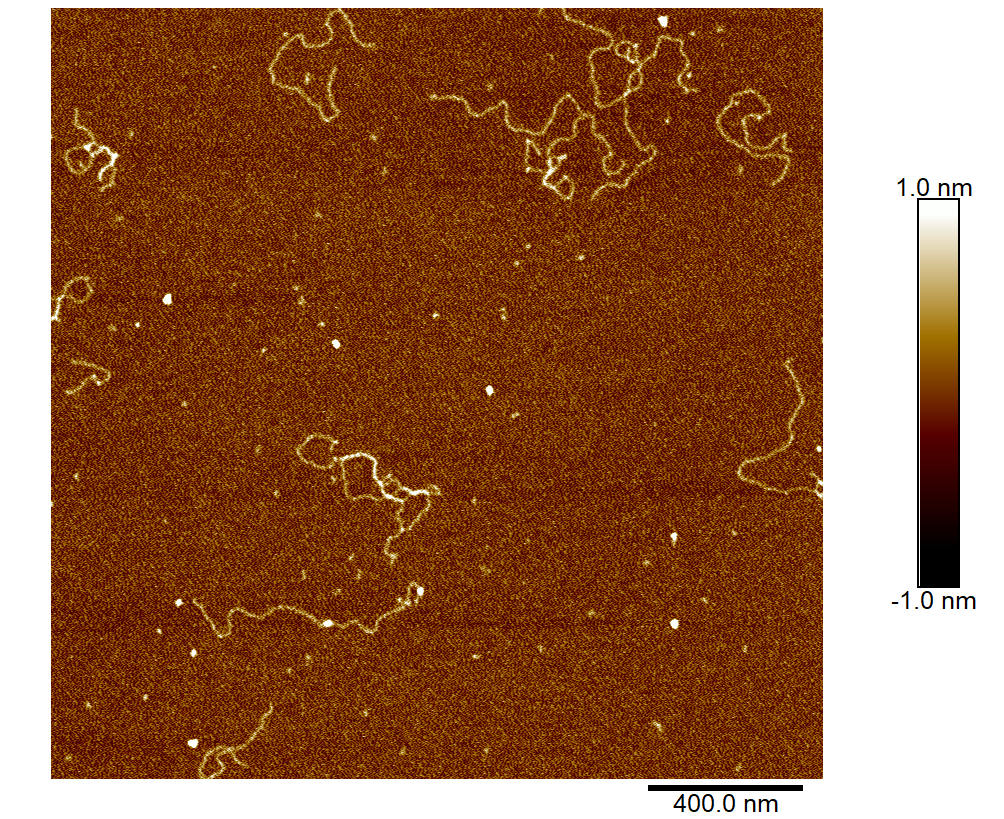

Supplement: Supplementary file 4 — Source data [file 41467_2024_49047_MOESM4_ESM.zip › Source Data/Rseponses to reviewers/linear_2um_.tif]

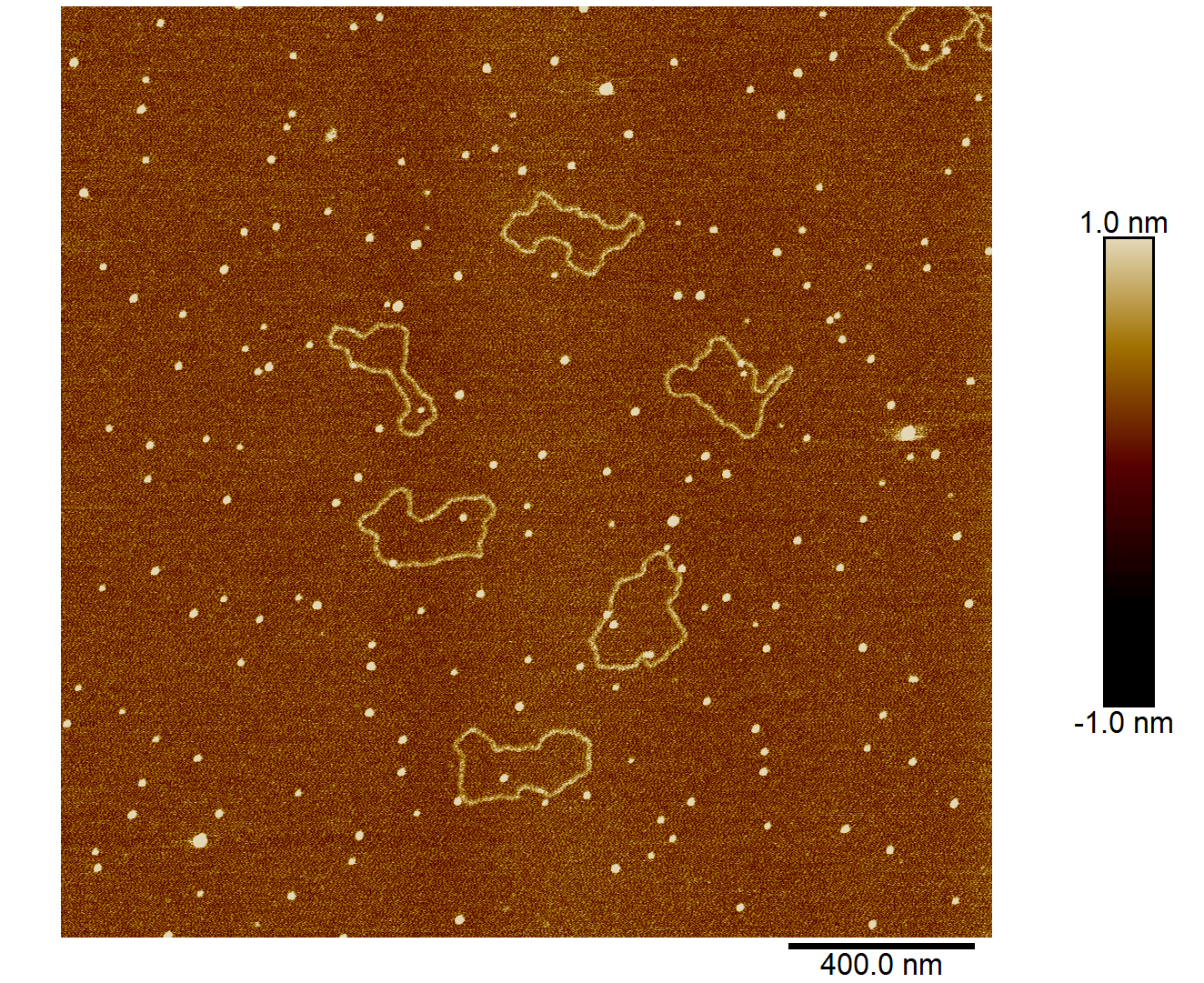

Supplement: Supplementary file 4 — Source data [file 41467_2024_49047_MOESM4_ESM.zip › Source Data/Rseponses to reviewers/relax2um.tif]

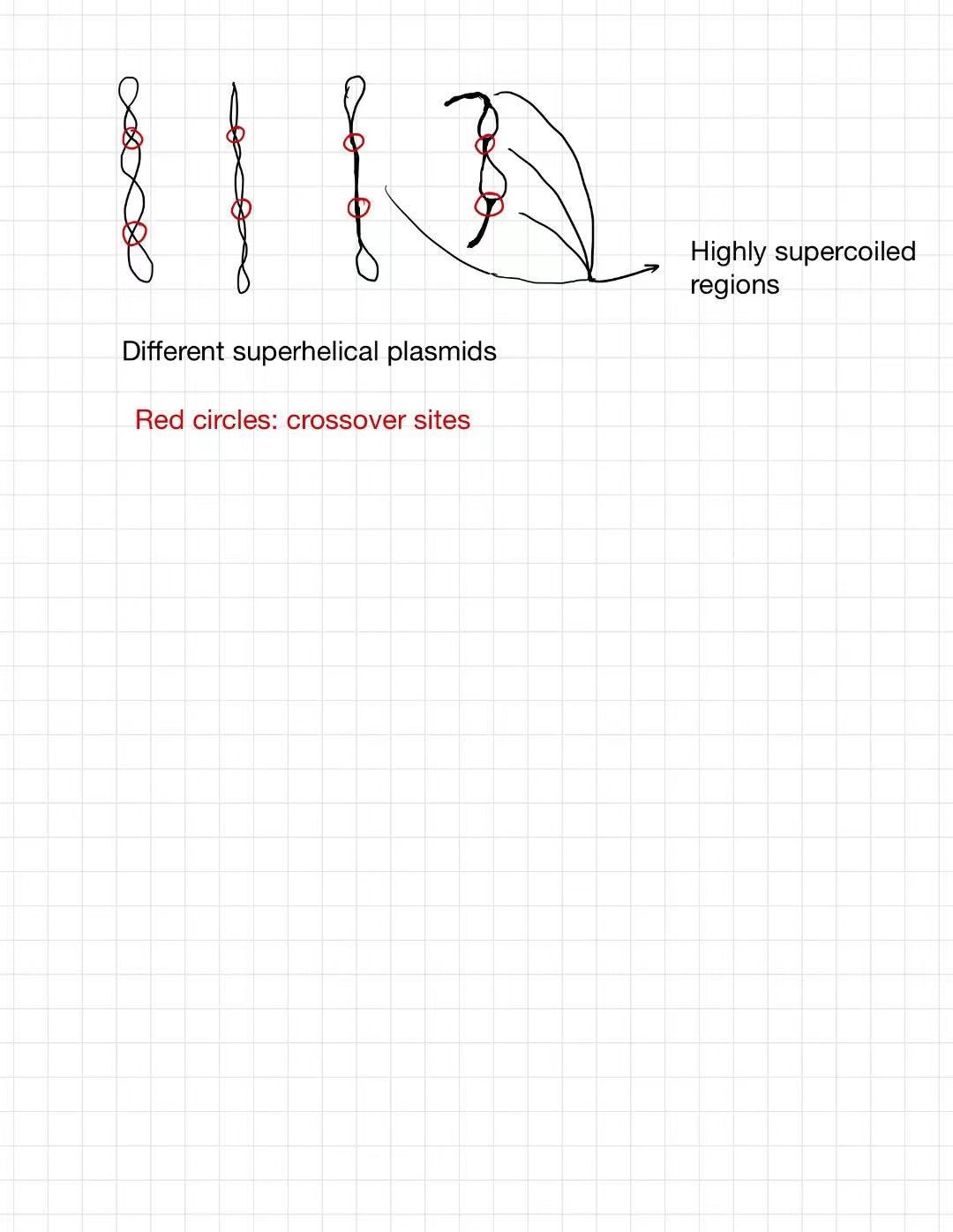

Supplement: Supplementary file 4 — Source data [file 41467_2024_49047_MOESM4_ESM.zip › Source Data/Rseponses to reviewers/supercoil.jpg]

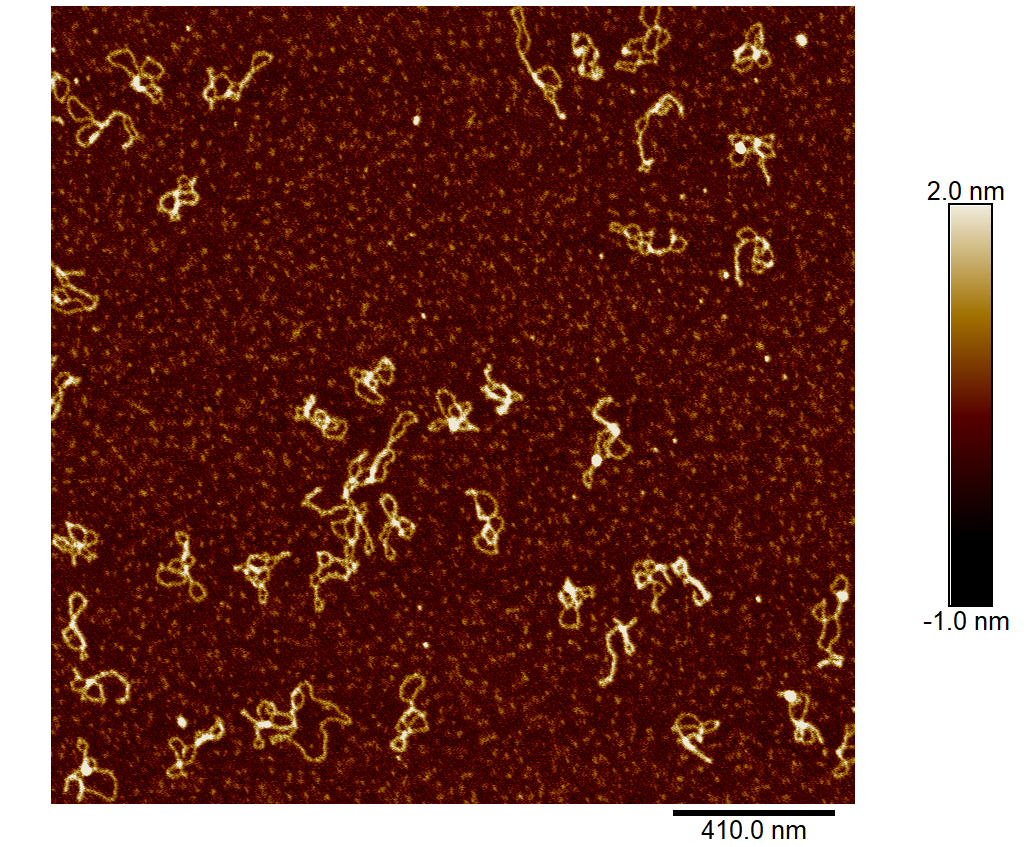

Supplement: Supplementary file 4 — Source data [file 41467_2024_49047_MOESM4_ESM.zip › Source Data/Rseponses to reviewers/supercoiled-2um.tif]

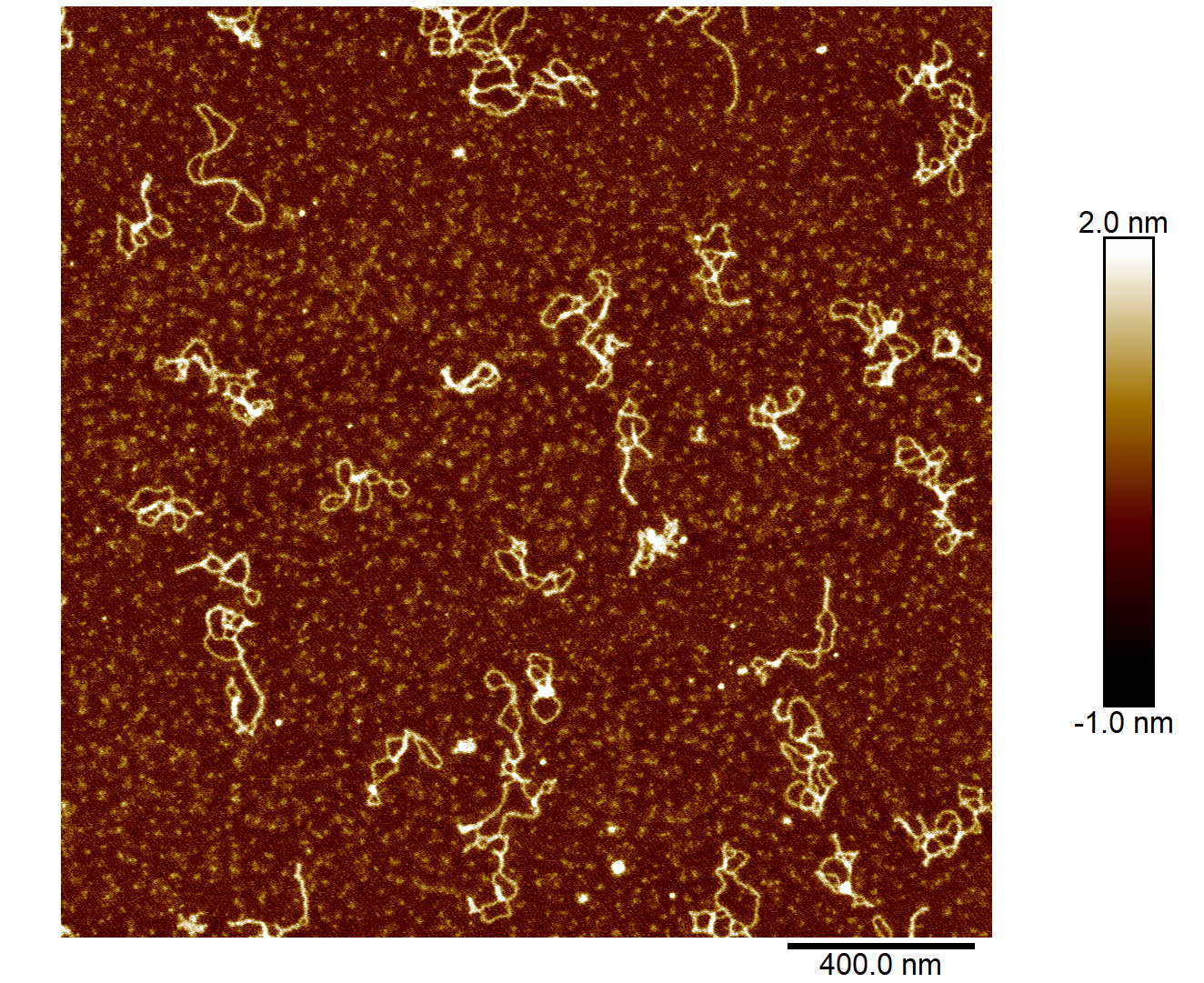

Supplement: Supplementary file 4 — Source data [file 41467_2024_49047_MOESM4_ESM.zip › Source Data/Rseponses to reviewers/supercoiled-2um1.tif]

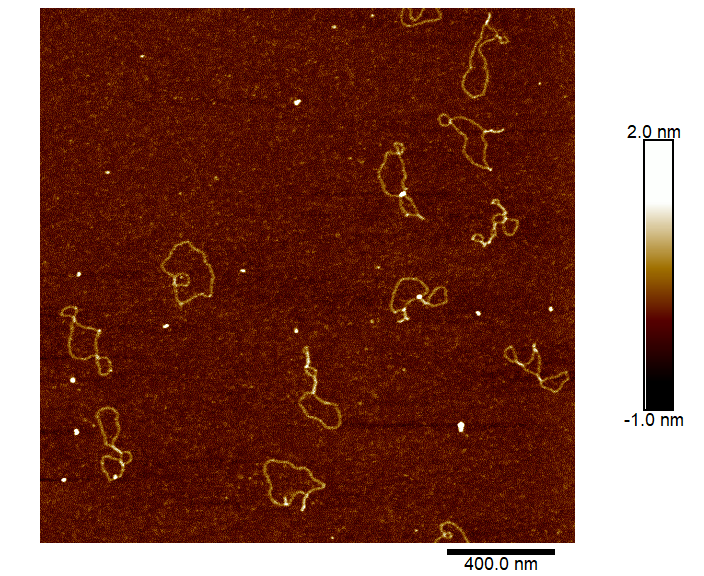

Supplement: Supplementary file 4 — Source data [file 41467_2024_49047_MOESM4_ESM.zip › Source Data/Supplementary Fig 12b1 and d right.tif]

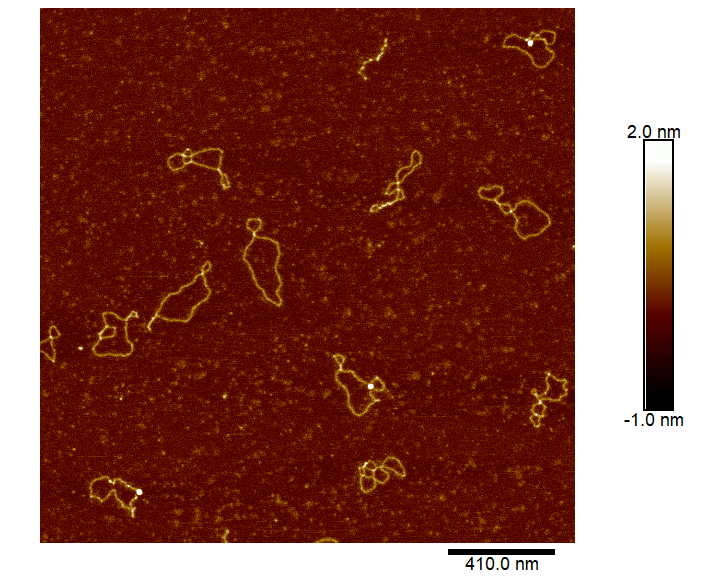

Supplement: Supplementary file 4 — Source data [file 41467_2024_49047_MOESM4_ESM.zip › Source Data/Supplementary Fig 12b2.tif]

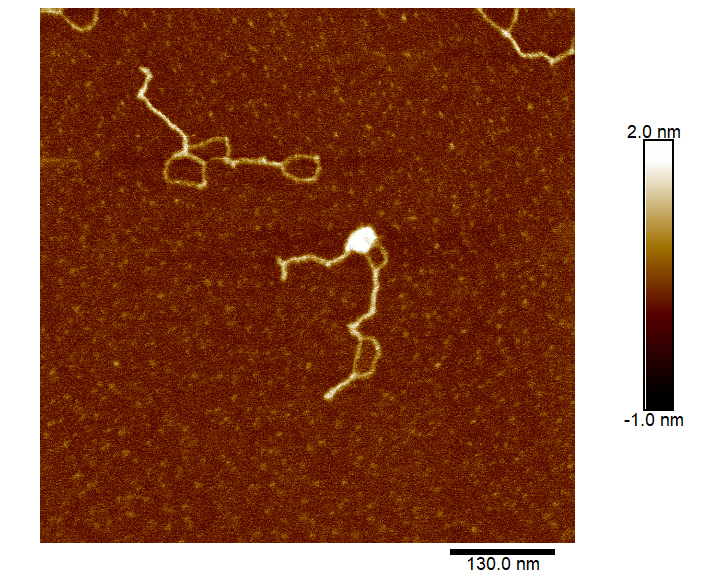

Supplement: Supplementary file 4 — Source data [file 41467_2024_49047_MOESM4_ESM.zip › Source Data/Supplementary Fig 12b3.tif]

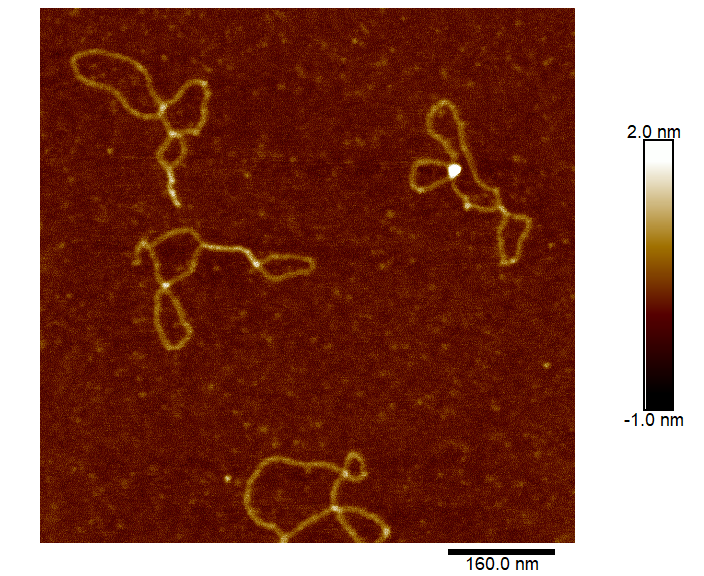

Supplement: Supplementary file 4 — Source data [file 41467_2024_49047_MOESM4_ESM.zip › Source Data/Supplementary Fig 12b4.tif]

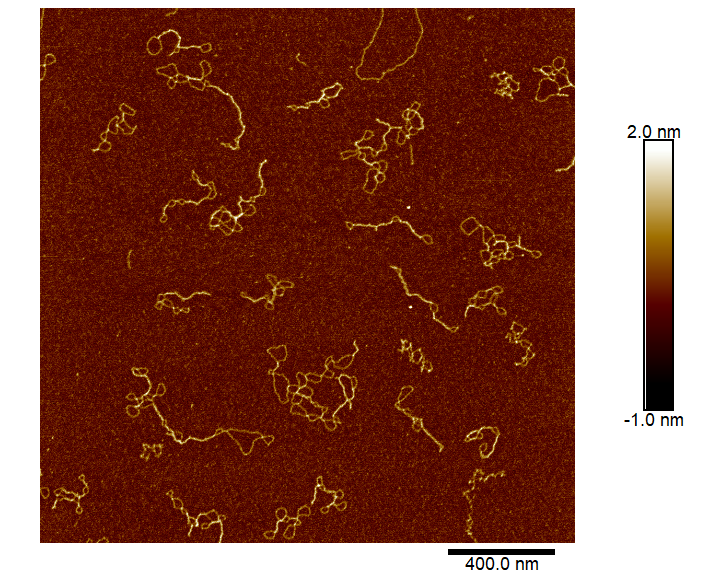

Supplement: Supplementary file 4 — Source data [file 41467_2024_49047_MOESM4_ESM.zip › Source Data/Supplementary Fig 12d left.tif]

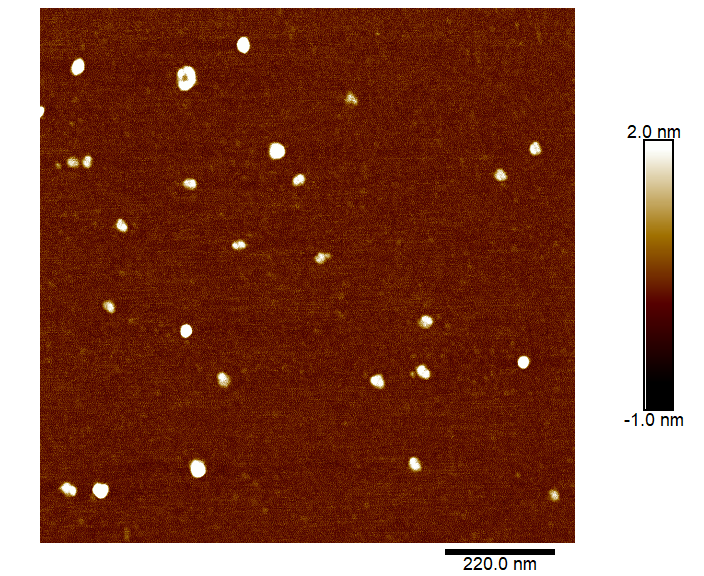

Supplement: Supplementary file 4 — Source data [file 41467_2024_49047_MOESM4_ESM.zip › Source Data/Supplementary Fig 12d middle.tif]

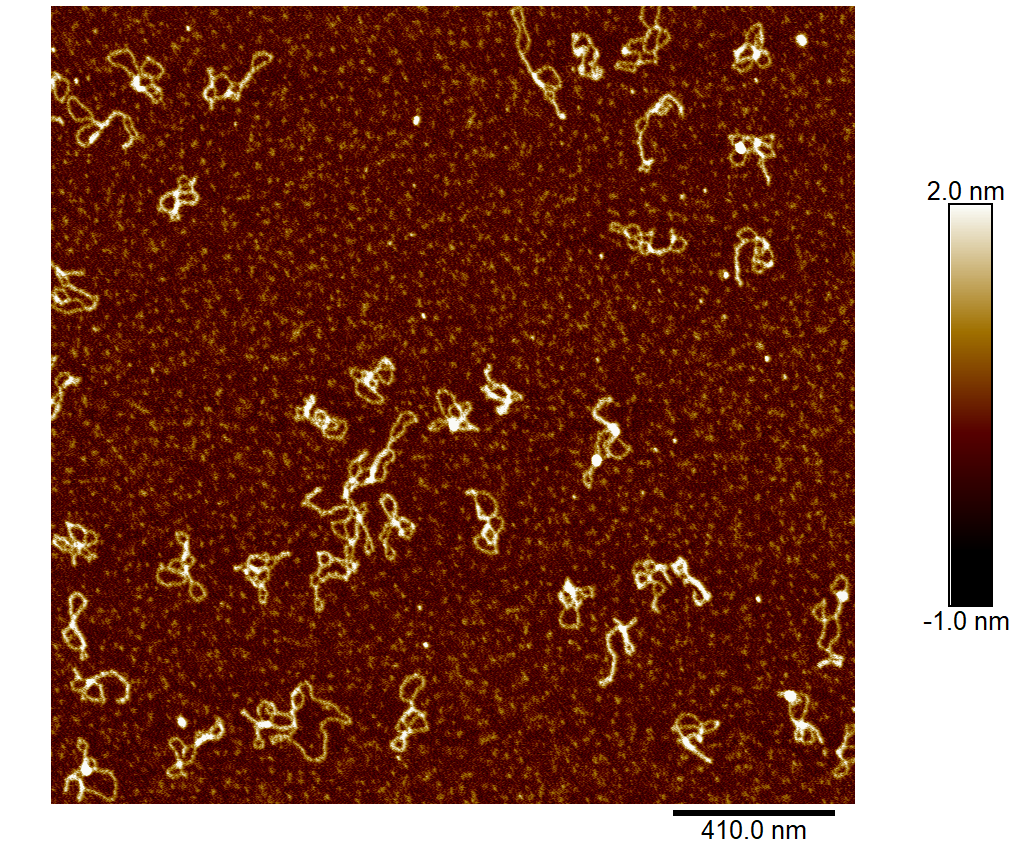

Supplement: Supplementary file 4 — Source data [file 41467_2024_49047_MOESM4_ESM.zip › Source Data/Supplementary Fig12a.tif]

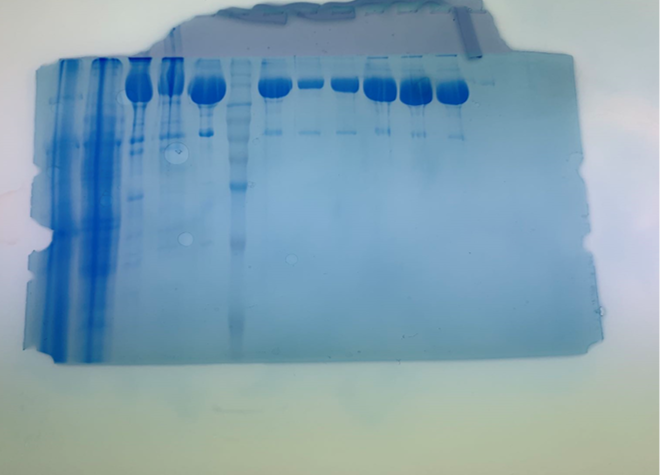

Supplement: Supplementary file 4 — Source data [file 41467_2024_49047_MOESM4_ESM.zip › Source Data/Supplementary Fig1a.png]

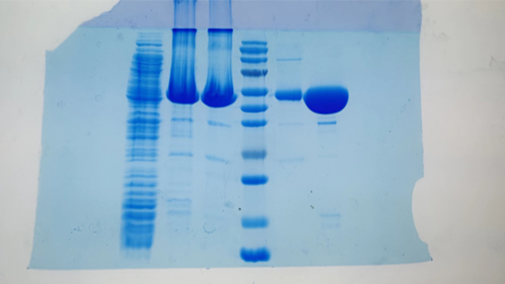

Supplement: Supplementary file 4 — Source data [file 41467_2024_49047_MOESM4_ESM.zip › Source Data/Supplementary Fig1b.png]
